# Supplementary material for: Correlating activities and defects in (photo)electrocatalysts using in-situ multi-modal microscopic imaging
Source: Nat Commun. 2024 May 9;15:3908. doi: 10.1038/s41467-024-47870-9 (PMC11082147; doi:10.1038/s41467-024-47870-9)
Supplement: Supplementary file 1 — Supplementary Information [file 41467_2024_47870_MOESM1_ESM.pdf]

Correlating activities and defects in (photo)electrocatalysts using in-situ multi-modal microscopic imaging

[1,2,3]Camilo A. Mesa‡, [1,4,5]Michael Sachs, [2,6]Ernest Pastor, [7]Nicolas Gauriot, [7]Alice J. Merryweather, [8]Miguel A. Gomez-Gonzalez, [8]Konstantin Ignatyev, [2]Sixto Giménez, [7]Akshay Rao, [1,9]James R. Durrant and [7,10,11]Raj Pandya✉

[1]Department of Chemistry and Centre for Processable Electronics, Imperial College London, London, W12 0BZ, United Kingdom

[2]Institute of Advanced Materials (INAM) Universitat Jaume I 12006 Castelló Spain

[3]Sociedad de Doctores e Investigadores de Colombia, Grupo de Investigación y Desarrollo en Ciencia Tecnología e Innovación - BioGRID, Bogotá 111011, Colombia

[4]SLAC National Accelerator Laboratory, Menlo Park, CA, USA

[5]PULSE Institute, SLAC National Accelerator Laboratory, Stanford University, Stanford, CA, USA

[6]CNRS, Univ Rennes, IPR (Institut de Physique de Rennes) - UMR 6251, F-35000 Rennes, France

[7]Cavendish Laboratory, University of Cambridge, J.J. Thomson Avenue, CB3 0HE, Cambridge, UK

[8]Diamond Light Source Ltd., Harwell Science and Innovation Campus, Didcot, Oxfordshire, OX11 0DE, United Kingdom

[9]Department of Materials Science and Engineering, Swansea University, Swansea, SA2 7AX United Kingdom

[10]Laboratoire Kastler Brossel, ENS-Université PSL, CNRS, Sorbonne Université, Collège de France, 24 rue Lhomond, 75005 Paris, France

[11]Department of Chemistry, University of Warwick, Coventry, CV4 7AL, United Kingdom

‡Present address: Catalan Institute of Nanoscience and Nanotechnology (ICN2), CSIC, Barcelona Institute of Science and Technology, UAB Campus, 08193 Bellaterra, Barcelona, Spain

✉Correspondance to: rp558@cam.ac.uk

Supporting Information

Supplementary Note 1: Width of microstructural breaks in hematite film and limitations of EDX 3

Supplementary Note 2: Macroscopic photoelectrochemical performance and electrochemical efficiency calculations 4

Supplementary Note 3: Estimation of local 'fill factors'/local incident photon to current efficiency 6

Supplementary Note 4: Reproducibility tests in other regions of hematite film 9

Supplementary Note 5: Magnitude of ΔR/R signal depending on spatial location for hematite regions adjacent to, inside and away from microstructural cracks 10

Supplementary Note 6: Origin of double-peaked spectra 11

Supplementary Note 7: XANES and speciation study of hematite photoanodes 16

Supplementary Note 8: Raw Raman spectra in 'dark' and 'light' conditions with bias 18

Supplementary Note 9: Structural changes under light and bias 23

Supplementary Note 10: Sn concentration distribution across films and oxygen concentration of T1, T2 and T3 25

Supplementary Note 11: Correcting for differences in absorbance between different regions of sample 27

Supplementary Note 12: Magnitude of ΔR/R signal depending on spatial location for hematite regions with different thicknesses 29

Supplementary Note 13: The effects of carbon impurities 30

Supplementary Note 14: Pump wavelength and fluence dependence of transient microscopy results 35

Supplementary Reference 38

Supplementary Note 1: Width of microstructural breaks in hematite film and limitations of EDX

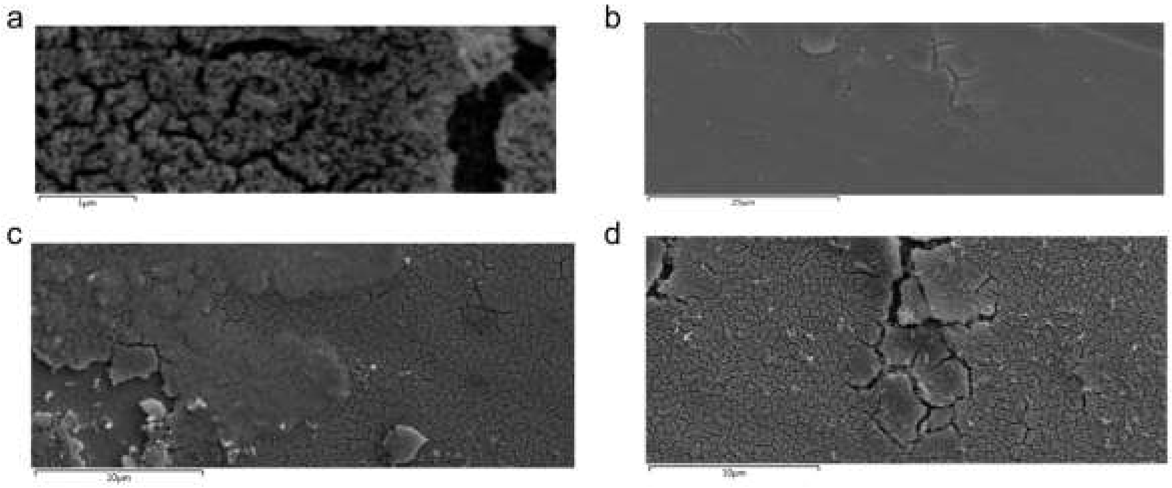

Supplementary Figure 1: a-d. SEM images of α-Fe_2O_3 film highlighting variation in crack sizes from 500 nm to 1500 nm in lateral width.

Using EDX to be more quantitative on oxygen vacancies concentration ([OV]) presents several challenges. Firstly, EDX (without a reference standard) is generally accepted to be a semi-quantitative method (but only under certain conditions). Quantification involves using the peak ratios to determine the relative proportion of components and then normalising them to 100% to account for all of a particular element. This method assumes the sample is flat (polished), homogeneous and 'infinitely' thick relative to the beam interaction volume. If these latter conditions are not met neighbouring components in the interaction volume will influence the results, i.e., there will be a topographic component. This will remain an issue to some extent even if external standards are used to calibrate the spectra. These so-called 'matrix corrections' are difficult in non-homogenous samples like ours because of the need to find representative 'background' areas e.g., of the same thickness, that are extremely homogeneous. Whilst other background subtraction methods exist, they tend to be less accurate. Furthermore, for quantitative EDX large accelerating voltages are often required to generate significant X-ray counts (up to 20 kV). We found these large voltages are particularly damaging to our samples. This is something that is key for us to avoid as we are performing correlated microscopy measurements. Finally, even if all the above corrections can be performed the accuracy of EDX tends to be about 1%. Given the variation in oxygen vacancy concentration we expect is about 1-2% we do expect that we would be at the limit of our ability to detect the oxygen concentration accurately with EDX. Indeed, it is for these reasons EDX is normally used for just compositional identification.

Whilst there will be a correlation between the O:Fe ratio from EDX and the oxygen content, EDX measurements will also be influenced by the atomic weight of the element trying to be detected. Light elements $Z<11$ are difficult to reliably quantify with EDX. Hence a direct comparison between EDX ratios and the oxygen vacancies measured by reflectivity measurements should be treated with caution. To calculate the concentration of oxygen vacancies, more complex techniques such as XPS could be used, however doing this with sub-10 μm resolution as would be needed for us remains challenging and well-beyond the scope of this work.

Supplementary Note 2: Macroscopic photoelectrochemical performance and electrochemical efficiency calculations

The local PEC performance of our samples is, at certain locations, higher than would be expected for a film that was prepared without high-temperature annealing.

Performing macroscopic I-V curve measurements (cm diameter beam spot, 1 sun white light illumination) across several films/locations we find current densities at 1.6 V vs RHE between 0.9 and 1.1 mA cm^{-2} depending on the location, with an average value of 0.97 mA cm^{-2} (see **Supplementary Figure 2** for non-local/macroscopic I-V performance curve). Based on the work of Zandi and Hamann[1] we might expect the non-locally measured current density at 1.5 V_{RHE} to be lower at ~0.6 mA cm^{-2} for such a low-temperature annealed film. For an annealed sample it should be around ~0.8 mA cm^{-2} based on this reference. However, these values vary in the literature with Mesa *et al.*[2], reporting much higher current densities for such low temperature annealed samples around 1.1 mA cm^{-2}. This suggests that our (macroscopic) I-V response is not necessarily unusual compared to the literature, but that the films we examine may not be as defective as a traditional, solovothermal preparation-based, defect rich, films.

Moreover, the fact that the local I-V response (0.7 to 1.3 mA cm^{-2} at 1.5/1.6 V vs RHE) lies above and below the range of the microscopic one, further supports the claim that there are regions of the film that locally can perform as well as ones that were annealed at higher temperatures, i.e., not all microscopic defects are detrimental to the performance.

We also note that our onset potentials are more in-line with low temperature annealed films at between 0.8 and 1.1 V vs RHE compared to the 0.6/0.7 V vs RHE that would be expected for annealed films. It is additionally important to bear in mind that as we are comparing relative current density/onset potentials between film locations, as opposed to absolute values, any discrepancies do not actually affect the conclusions of our work.

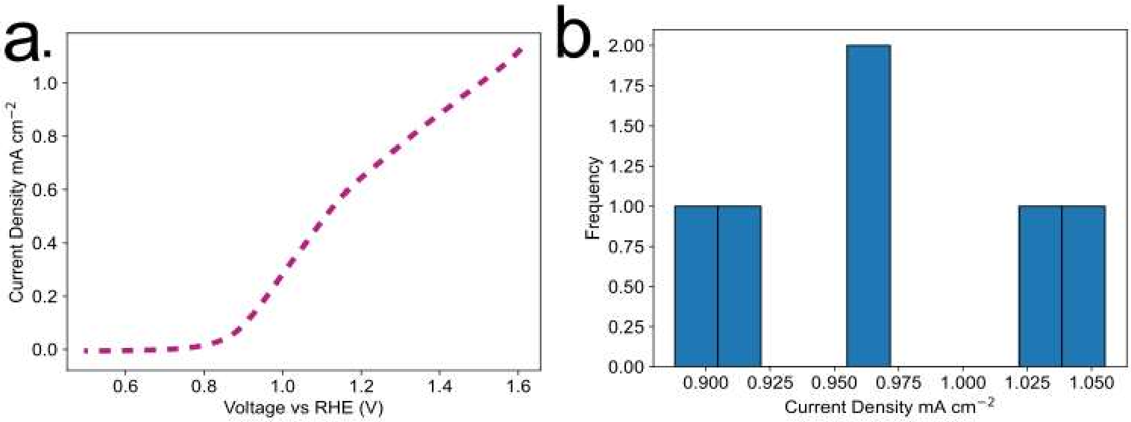

Supplementary Figure 2: a. Macroscopic I-V curve of typical haematite electrode measured in this work at 1 sun illumination. **b.** Photocurrent at 1.6 V vs RHE of identically prepared (solvothermal, low-temperature annealed) haematite electrodes.

Finally, we remark there may be nanoscale morphology in our films, that we cannot resolve, which may be playing a role. Indeed, the nanorod morphologies that are typically expected to be present in the types of films we measure are only visible at the sub-100 nm lengthscale (~50 nm wide nanorods). As in our work we are focussing on *microscale* (1 µm and above) structure that is accessible with optical and X-ray techniques we cannot comment on the impact of such features. Higher-resolution SEM images (500 nm resolution) of the films confirm that such features are present (see **Supplementary Figure 3**).

We note that even under the same preparation conditions variations in the performance haematite can emerge. Hence, we do not believe it is surprising that our film does not behave exactly as all previous reports (see variation in photocurrent between ref 1 and 2 where the same synthetic route is reported but the maximum photocurrent at 1.6 V vs RHE varies by almost 0.5 mA cm^{-2}).

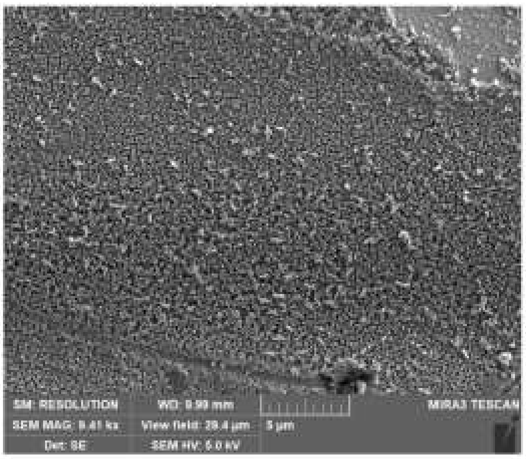

Supplementary Figure 3: Scanning electron microscope (SEM) image of an α-Fe_2O_3 photoelectrode studied in this work. The top part of the figure shows a 15 × 20 µm area SEM image of the electrode. Sub-micron nanorod-like features can be seen to emerge on the surface as bright (white) line-like objects. The bottom strip details the SEM parameters including the scale bar and SEM system name which is a MIRA3 TESCAN. Other abbreviations: MAG – magnification; WD – working distance; SEM HV – electron microscope imaging (high) voltage; Det:SE – secondary electron detector.

Supplementary Note 3: Estimation of local 'fill factors'/local incident photon to current efficiency

We agree with the reviewer that it is important to make our analysis as quantitative as possible. We have now estimated both local 'fill factors' (FF) as well as local incident photon to current efficiencies[3] (IPCE) for the film in the different spatial regions considered. For the fill factor, we define the thermodynamic oxidation potential of water (1.23 V vs RHE) as the 'short-circuit' conditions. Given that the performance of a solar cell (**Supplementary Figure 4a**) is normally characterised as the maximum power ($V_{Max} \times I_{Max}$) divided by the maximum power point, MPP ($V_{OC} \times I_{SC}$), we estimate equivalently the FF of our hematite photoelectrodes at 0 V of overpotential (η), i.e., the thermodynamic potential of the OER (1.23 V vs RHE). Thus, the maximum photoelectrochemical power that can theoretically be achieved by our hematite electrodes is taken as $\eta \times I_{Meas}$ see **Supplementary Figure 4b**, left panels. We note for η we take the onset voltage (when the current density exceeds 0.1 mA cm^{-2}), and for I_{Meas} we use the current density at 1.23V. Whereas the maximum power that actually is achieved is $\eta_{Onset} \times I_{@\eta=0}$, i.e., the maximum in a plot power density *vs* voltage (**Supplementary Figure 4b**, right panel**)**. This method of calculation is supported by the work of Hodes[4].

On the other hand, the IPCE at 1.23 V vs RHE can be determined by the ratio ($I_{Meas@1.23V}$/ $I_{Theor@1.23V}$) between the measured photocurrent at 1.23 V vs RHE and the maximum achievable photocurrent density of our photoelectrode. This maximum photocurrent density is calculated using the absorption spectra of our hematite photoelectrode as follows:

(i) The local absorption spectra at each point in the film are obtained by combining the reflection spectra measurements from **Figure's 4** and **6** of the main text with transmission spectra measurements at the same location.

(ii) The local absorption is multiplied by the total solar flux under AM1.5 solar irradiation (normalised solar spectrum). However, as in our measurements of photoelectrochemical activity we are using a laser at 532 ± 15 nm, we only take the solar spectrum and absorption between 515 and 545 nm. In this way we can obtain the theoretically absorbed photon flux at each local point on the film examined.

(iii) The absorbed energy is divided by the photon energy at each wavelength to give the maximum number of photo-generated electrons and holes.

(iv) Using the above value and the integration time at each point in the local photocurrent measurements we can obtain the maximum possible photocurrent at each spatial location.

(v) Comparing the above theoretical current density with the photocurrent density measured at the local water oxidation potential allows us to obtain a local FF/IPCE for each location in the film.

The above procedure is summarised in **Supplementary Figure 5**. Although this analysis is simplified e.g., does not account for dark currents it allows a semi-quantitative understanding to be obtained. Following the above procedure, we find a theoretical maximum current for our films of 3.07 ± 0.1 mA cm^{-2}.

Based on the above value, examining the CR, ACR and FL regions as above we find the local FF values vary between of 0.36 and 0.21 in the order ACR>FL>CR (**Supplementary Figure 6a**). For T1, T2 and T3 the FF ranges between 0.19 (T1) and 0.33 (T3) (**Supplementary Figure 6b**); errors are 0.06. The IPCE varies between 10% and 22% in the order ACR>FL>CR (**Supplementary Figure 6c**). For T1, T2 and T3 the FF/IPCE ranges between 13% (T1) and 18% (T3) (**Supplementary Figure 6d**); errors are ±4%. Interestingly the trend in FF/IPCE of T3>T2>T1 is more evident than for the photocurrent,

suggesting indeed that subtle variations in the film thickness and associated vacancies can play a role in the water oxidation kinetics.

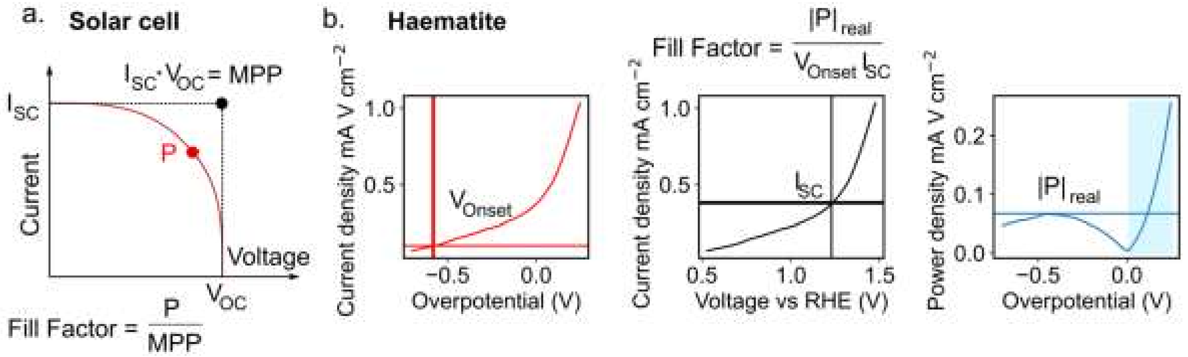

Supplementary Figure 4: a. Schematic of how performance is estimated for solar cells. **b.** Outline of how we quantitatively estimate the fill factor of our haematite photoelectrodes. To estimate the theoretical power density that can be achieved we can take a plot of the overpotential *vs* current density from which we estimate the onset overpotential (when the current density exceeds 0.1 mA cm^{-2}). We can also determine the short circuit current from the point at which the voltage vs RHE reaches 1.23 V. Multiplying these together gives us the maximum power performance. Multiplying the current density and voltage and plotting this *vs* overpotential then allow us to estimate $|P|_{real}$ which is the maximum power actually achieved. From these two quantities a fill factor can be estimated.

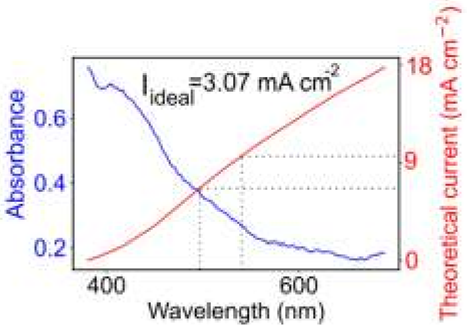

Supplementary Figure 5: Ensemble absorption spectrum and cumulative sum of theoretical current density as a function of wavelength based on film absorption and solar spectrum. The dotted line shows the region of the cumulative current density (and film absorbance) that contributes in the wavelength range we illuminate our electrodes in.

Ensemble absorption spectrum and cumulative sum of theoretical current density as a function of wavelength based on film absorption and solar spectrum. The dotted line shows the region of the cumulative current density (and film absorbance) that contributes in the wavelength range we illuminate our electrodes in.

Additionally, the incident photo to current efficiency (IPCE). This term is effectively a measure of a photoelectrodes external quantum efficiency[3]. For the best α-Fe_2O_3 electrodes e.g., for ones prepared using atmospheric pressure chemical vapour deposition with passivation SnO_2 layers, IPCE values reach about 22% at 532 nm (and >1.4 V vs RHE)[3]. This shows that locally our defective electrodes do have morphologies present that can give rise to IPCEs not only above that of the native film but also close to that of samples where the film preparation is optimised for performance. In other morphologies (which dominate throughout the film) the IPCE we obtain is low. The dominance of these low IPCE regions means the overall PEC of the sample is quite low. (A similar argument can be made based on the fill factors[4,5].

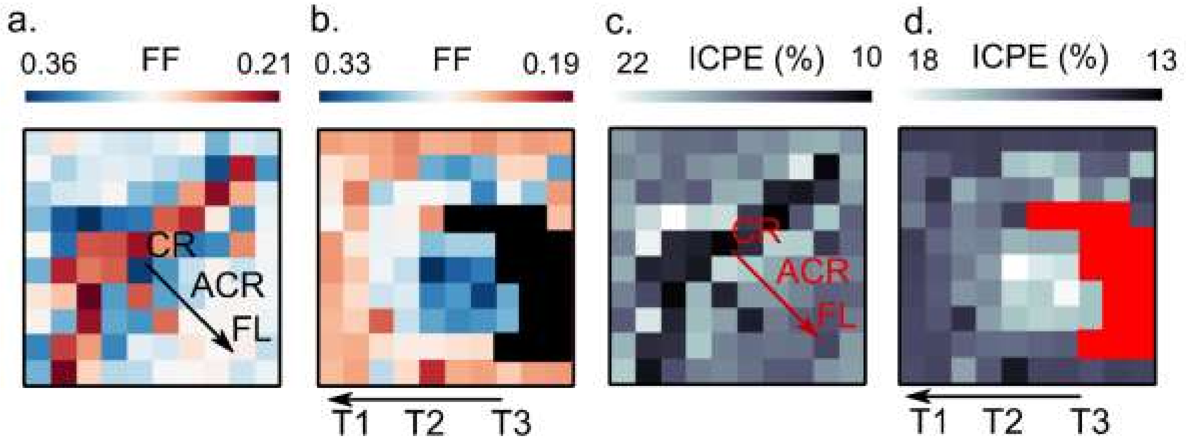

Supplementary Figure 6: a-b. Map of fill factor (FF) *versus* spatial location for CR, ACR and FL regions displayed in **Figure 3** of the main text and T3 to T1 regions displayed in **Figure 5** of the main text. **c-d.** Map of incident photon to current efficiency *versus* spatial location for CR, ACR and FL regions displayed in **Figure 3** of the main text and T3 to T1 regions displayed in **Figure 5** of the main text. In both FF and IPCE values there are clear trends in the behaviour similar to the photocurrent density. The black/red region is the substrate and no calculation is made here.

Several publications relate oxygen vacancies with surface states that participate in the OER mechanism[6–8]. However, more recent kinetic analysis involving correlating the measured optical absorption of photogenerated holes with the measured rate of OER, suggests that oxygen vacancies seem to have a more significant impact in recombination than in the surface reaction process[9]. In this paper, the rate determining step (RDS) of the OER was studied in 4 different hematite photoanodes, with different optical and electronic properties (including doping density and oxygen vacancies). Kinetic analyses revealed a 3rd order process with respect to surface hole density for all the samples studied, suggesting that the RDS is achieved when 3 photogenerated holes are accumulated at the reaction centre[10]. Additionally, in these studies, experiments at different applied potentials (i.e., different filling states of the oxygen vacancies) revealed the RDS follows also a 3rd order of reaction, which suggests that the reaction mechanism does not change, regardless the amount of oxygen vacancies or their filling state. Consequently, as we discuss in the main text while oxygen vacancies can participate in the surface reaction, as coordination sites or to accumulate surface holes, we believe their role is more important in recombination losses.

To determine further the role of surface kinetics and the OER mechanism local transient photocurrent (TP) mapping could be performed. However, performing such a measurement locally remains challenging. The main problem is that analysis of TP measurements relies on the excitation being a small perturbation. Whilst this is true when performing macroscopic measurements, in the case of local focussed excitation, a relatively large excitation density is required to obtain a measurable signal. The interpretation used for macroscopic signals is no longer valid and analysing the data requires a new framework to be built. We note that for the transient reflection microscopy we perform the issue of high excitation densities is less of an issue for interpretation of the kinetics (especially as we stay at the limit of the linear excitation regime; see **Supplementary Note 14**). Furthermore, empirically (at the ensemble level) this all-optical measurement is easier to obtain a signal from at low excitation densities than TP.

Another point that should be noted is that in the few cases when transient photocurrent mapping has been performed it has been on relatively homogeneous samples (2D materials, photovoltaic thin-film blends[11,12]). This is because optical scattering can influence the measured transients. This would be a further cause for concern when trying to perform such measurements on our samples[11].

Supplementary Note 4: Reproducibility tests in other regions of hematite film

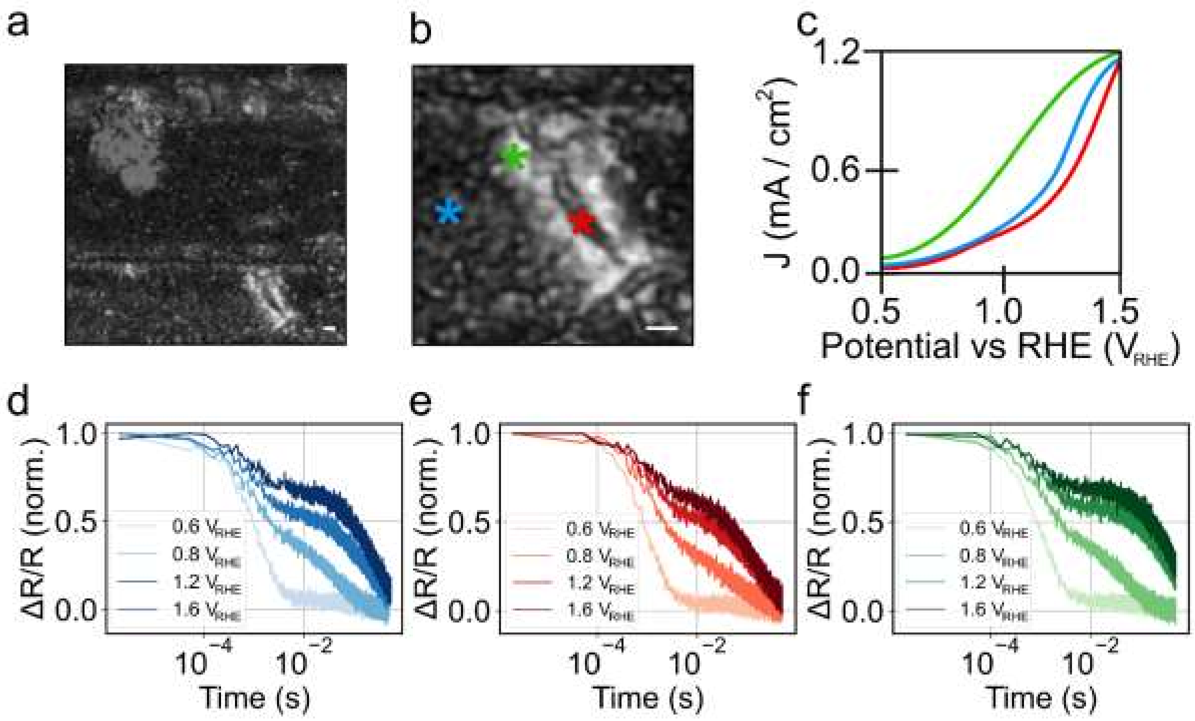

Supplementary Figure 7: a. Bright-field image of hematite photoelectrode. Scale bar is 1 µm. **b.** Zoomed in image of crack (red asterisk) on photoelectrode and region adjacent (green asterisk) to it, as well as native film (blue asterisk). Scale bar is 1 µm. **c.** Photocurrent from regions marked in b. Similar behaviour to that described and explained in the main text is observed for cracks, regions of the film adjacent to the crack and the native film. **d-f.** Normalised ΔR/R kinetics from three regions marked in **b**. The trend in kinetics both with potential and spatial location is similar to that described in the main text **Figure 3** and **4**.

Supplementary Note 5: Magnitude of ΔR/R signal depending on spatial location for hematite regions adjacent to, inside and away from microstructural cracks

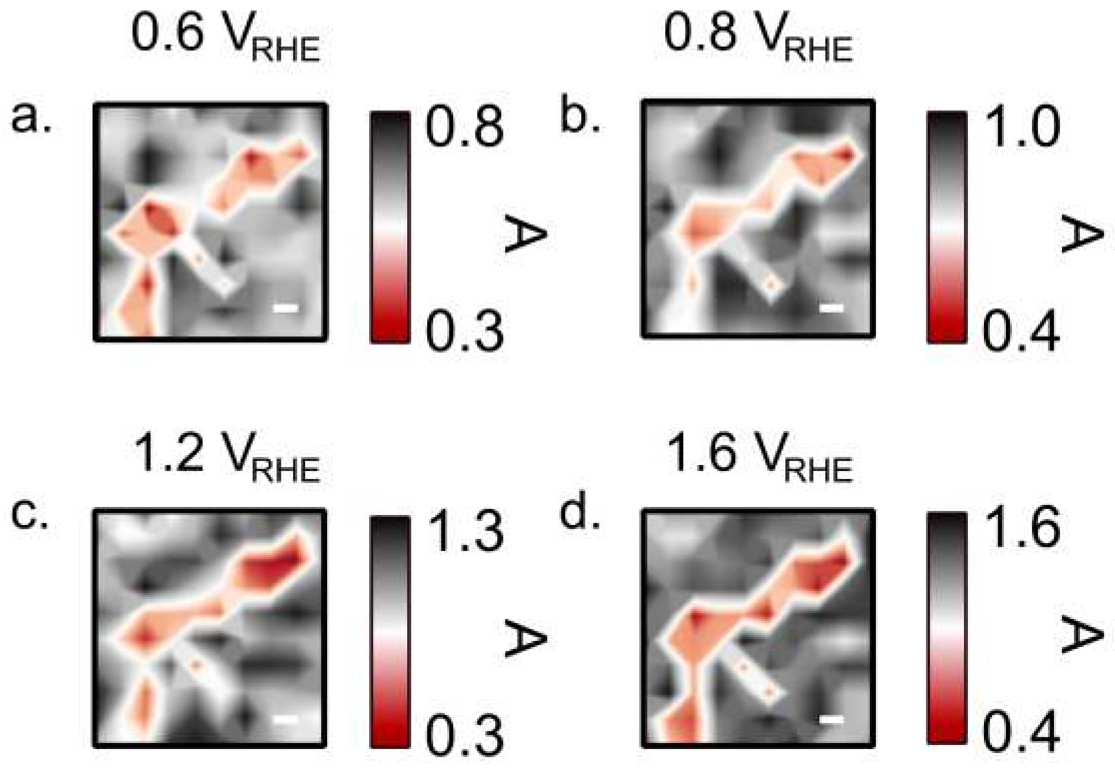

Supplementary Figure 8: a-d. Spatial maps of magnitude of ΔR/R kinetic (A) as a function of spatial location at potentials of 0.6 to 1.6 V_{RHE} for regions shown in **Figure 3** and **4** of the main text.

Supplementary Note 6: Origin of double-peaked spectra

Generally assigning new absorption peaks unequivocally in any material is exceptionally challenging, often involving consensus at the community level, both experimentally and theoretically. Here we have focussed on performing experiments that might provide some insight on the species responsible and ruling out other sources.

Surface states: A double peaked absorption spectrum with peaks separated by 120 nm (460 nm and 580 nm peaks) which grow in with bias were observed by Klahr and Hamann[13]. In this case the response was linked to the absorption of Fe=O and Fe-OH surface states. As this peak separation is significantly larger than that which we observe, we can rule out this contribution.

The above work did also report a peak splitting of the central absorption peak at 580 nm into two peaks at 560 ± 5 and 570 ± 5 nm. This is narrower than we observe and was only seen to occur in certain potential ranges. Calculations by Snir and Toroker[14] demonstrated that these peaks arise from *OH and *O surface species which we can also rule-out.

High oxidation Fe species: The absorption spectra reported for Fe(IV), Fe(V), and Fe(VI) species in solution in the 400-700 nm spectral region are as follows: Fe(IV) shows a broad peak at 420 nm, Fe(VI) has a broad peak at 510 nm, Fe(V) has a main peak at 400 nm and a second peak at 500 nm. None of these peaks match with our observations and hence we can likely rule out their role in giving rise to the double-peaked spectrum we observe[15].

Kinetics of two absorption peaks: In **Supplementary Figure 9** we show spectrally resolved transient absorption measurements (without bias) on several locations of the haematite films. Interestingly in certain regions of the film a double peaked feature appears in the transient absorption spectra (355 nm pump; 200 nJ; 200 fs time resolution), with the peak centres closely matched with those of the two ΔR peaks detailed in the main text. The transient absorption spectra report mainly on the photoinduced absorption (PIA) bands of α-Fe_2O_3, whereas the in the ΔR spectra we are examining ground state absorption. The two are hence not directly comparable. Indeed, because the ground state bleach of hematite is particularly short (<1 ps) it becomes obscured by the PIA. However, there will be an underlying ground state bleach kinetic within the transient absorption spectra which will reflect the lifetime of the two states we are sensitive to in the ΔR spectra. Furthermore, the structure we observe in the PIA bands can also be created by an underlying absorption structure that is reflected in the bleach, i.e., is not from two distinct PIAs. Altogether the above makes it challenging to directly assign the transitions we are observing in the transient spectra and correlate them with the data we see in the ΔR spectra. But: (i) the uniform decay kinetics across 500-700 nm wavelengths suggest the states responsible for the two peaks have very similar lifetimes and (ii) may be related to some underlying states present in certain regions in the ground state of the material (i.e., present without bias).

We note that performing spectrally resolved transient reflection mapping remains technically challenging so we cannot correlate the spectra in **Supplementary Figure 9** with exact regions of the film. However, to further verify point (i) in the above we repeated transient reflection measurements in the T3 region detailed in **Figure 5** of the text at 540 nm and 590 nm (at 1.6 V vs RHE). Identical decay kinetics are found to those previously detailed at both wavelengths (**Supplementary Figure 10**).

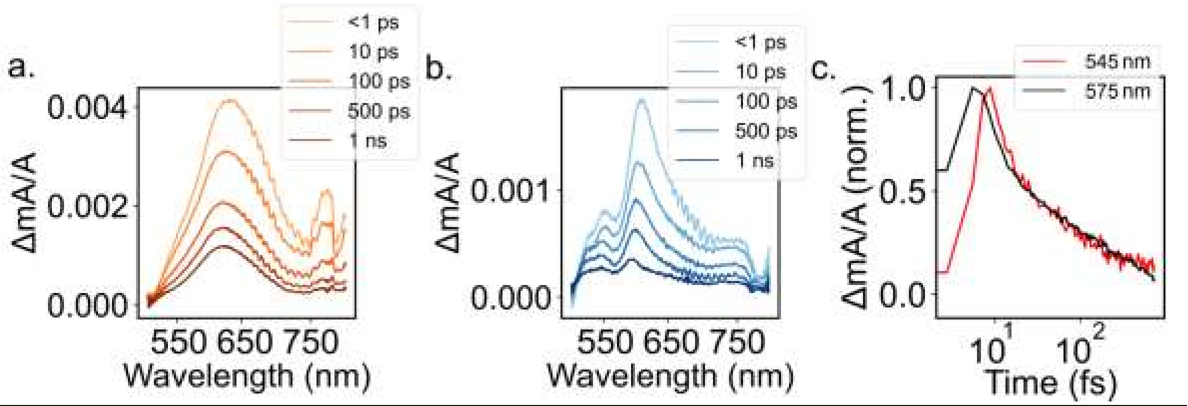

Supplementary Figure 9: a-b. Spectrally resolved transient absorption spectra of haematite thin films measured in this manuscript at location showing single peaked photoinduced absorption bands (a) and double peaked bands. **c.** Kinetics at 545 nm and 575 nm from transient absorption spectra in b.

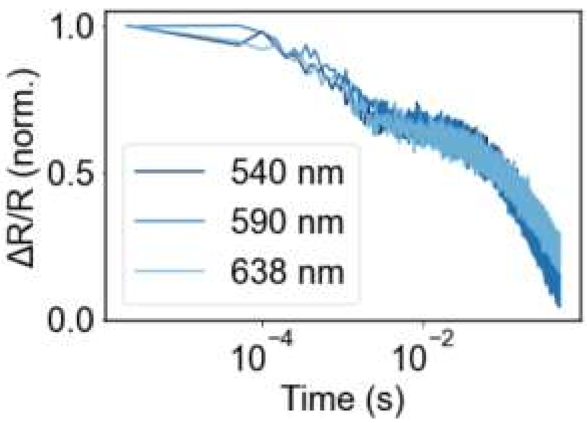

Supplementary Figure 10: Transient reflection kinetics of T3 region detailed in **Figure 5** and **6** of main text, measured at 540 nm 590 nm and 638 nm.

Polarizability and dipole moment: We performed electroabsorption (EA) of measurements on the haematite films to understand how the transitions reported in the single and double peaked ΔR spectra are sensitive, if at all, to electric fields, or if they can be revealed more clearly by the presence of a field. To create devices for electrabsorption spectroscopy the FTO glass was coated with 70 nm of Al_2O_3, haematite was deposited as in the main text, 70 nm of Al_2O_3 was then placed atop of the film with a 15 nm Cr/Au semi-transparent contact pad. Electroabsorption measurements were performed in a transmission geometry using the same setup as previously detailed in ref[16]. All measurements were performed at a fixed field of 600 kV/cm as this produced sufficient signal-to-noise ratios. Most simply the EA signals can be modelled as a combination of linear:

$$\Delta\alpha = -\frac{1}{2}\Delta p F^2 \frac{d\alpha}{dE}$$

where $\Delta\alpha$ is the EA signal, $\frac{d\alpha}{dE}$ the first derivative of the absorption spectrum, Δp the change in polarizability and F the electric field, and non-linear terms:

$$\Delta\alpha = -\frac{1}{6}\Delta\mu^2 F^2 \frac{d^2\alpha}{dE^2}$$

where $\Delta\mu$ is the change in dipole moment and $\frac{d^2\alpha}{dE^2}$ the second derivative of the absorption spectrum. While more sophisticated models exist to analyse the electroabsorption data for our exploratory measurements we stick to this well-applied model[17]. Applying this analysis model to the electroasbsoprtion spectra shown in **Supplementary Figure 11** we find relatively good agreement between the EA and the weighted derivative spectra. In other words, any additional band-edge transitions do not become more clearly visible in EA. This may be due to their insensitivity to the field, weak oscillator strength/forbidden selection rules or limited sensitivity of the technique itself. Nonetheless the EA spectra do allow us to estimate that in α-Fe_2O_3 the Fe *d-d* band-edge excitons at 590 nm have a $\Delta\mu$ of 2.4 D and Δp of 1.5 Å^3 (35% fitting errors on both). This is in-line with rather localised electron-hole pairs with a charge-transfer like exitonic-character (from the large dipole moment) as would be expected for a material like α-Fe_2O_3. Finally, we remark that further analysis is required to fully characterise electric field effects of transitions in the material e.g., field-dependent measurements and new modelling, but these are beyond the scope of the current work.

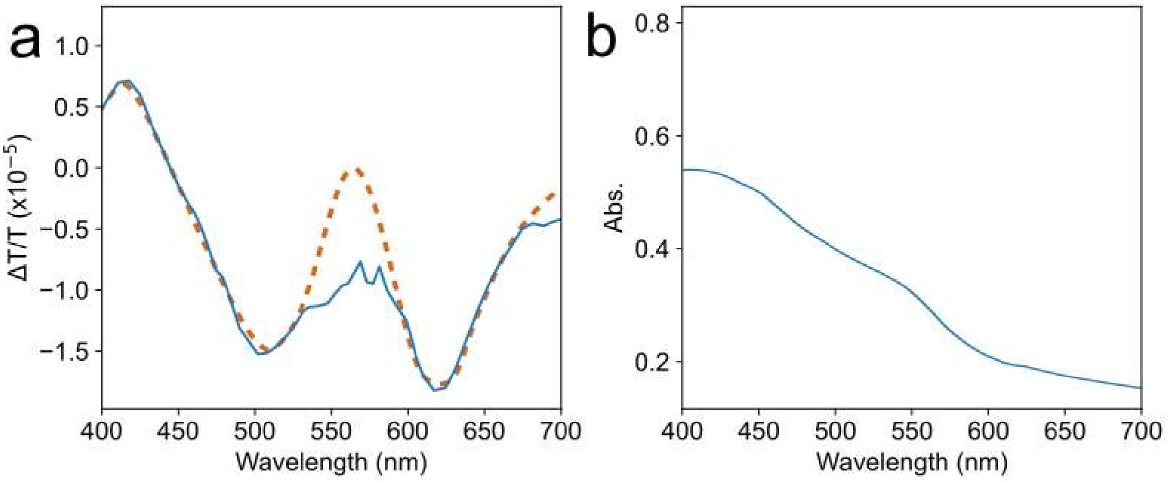

Supplementary Figure 11: Electroabsorption spectrum of α-Fe_2O_3 (solid blue line) with associated first and second derivative fit (orange dotted line). **b.** Corresponding film absorption.

<u>Theoretical basis for double peaked absorption:</u> Several theoretical works examine the absorption spectrum of hematite using *GW BSE* electronic structure calculations[18]. Interestingly, all works report the presence of two closely separated optical transitions, separated by 0.15 eV around the band-edge. These transitions are sometimes assigned to originating from the same or different states (direct and indirect transitions) and have a similar spacing to that observed in our work. Whilst the evolution of these transitions with bias has not been examined, they have experimentally been observed in the absorption spectrum of films with nanorod morphology, as we have[19]. We consequently can tentatively suggest that the two peaks we observe may be related to different hole transitions. We note given we observe similar decay times in the transient absorption spectra across the spectral range that is likely associated with these hole transitions, it is likely they originate from the same electronic state, i.e., are not necessarily distinct direct and indirect transitions.

To shed further light on where these transitions may arise from we can turn to the work of Marusak *et al.*[20]. Here, they measured the absorption spectra of α-Fe_2O_3 (110 crystal face) with high-sensitivity at zero bias and analysed the spectra using ligand-field theory. They also found two closely spaced transitions (albeit separated by 0.22 eV) and ascribed them to transitions between $^6A_1$ -> $^4A_1$ (high-energy transition) and $^6A_1$ -> ^4E (lower energy transition) symmetry states in the ligand field of the iron atoms, i.e., Fe (3d) valence band to Fe (3d) conduction band transitions. The energy separation of the transitions we observe matches well with both the above description and our spectra. The difference in intensity/presence between peaks in the voltage differential reflectivity spectra may then be a consequence of the selection rules and strength/presence of these transitions in a particular region of the film. In any case the work of Marusak *et al.* and ref[18] suggest that we are observing transitions between two states of the crystal field of the iron centres in hematite, i.e., two different hole species. These species appear to involve both similar electronic states and have a similar chemical behaviour, i.e., we can tentatively suggest they show the same general OER mechanism.

It is unclear what drives the presence of this double peaked spectrum in our films. For example, our T1 region which we suggest is rich in oxygen vacancies does not show this peak, whereas the similarly oxygen vacancy rich ACR region does. This suggests this behaviour is not linked only to the oxygen vacancy concentration/presence. Similarly, structurally there does not seem to be a consistent signature for this behaviour response e.g., the A_g/E_g mode intensity is high in T2/T3 but low in ACR. What is common to the appearance of this behaviour is high $t_{50\%}$ values, suggesting it is related to the underlying electronic states involved/accessible in the recombination/after photoexcitation. However, we believe commenting on this further is beyond the scope of this work.

Impurity phases: Other phases of hematite e.g., β-Fe_2O_3 and ε-Fe_2O_3 have band-edges at 1.9 eV and 1.35 eV, respectively. These are well away from that of the samples we examine and do not appear to contribute[21].

Faceting/nanostructuring: To rule out effects from nanostructuring/preferential faceting we have performed polarised absorption of films in ACR-like regions at 0.8 vs RHE examining the intensity of the two peaks as a function. (Fixed polarisation incoming light, varying polarisation in detection). We find no dependence on the detection polarisation, suggesting there is no preferential alignment in the microstructure of the films (**Supplementary Figure 12**).

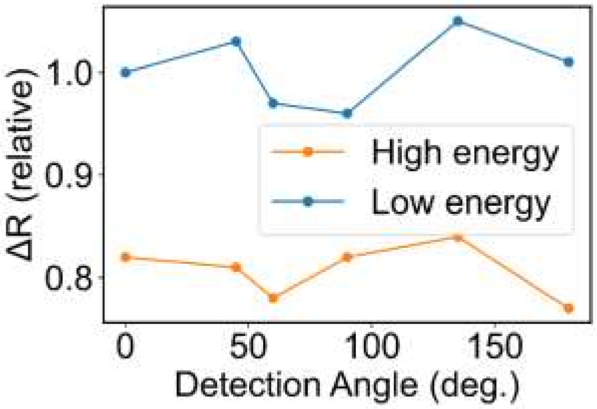

Supplementary Figure 12: Magnitude of change in reflectivity (maximum normalised intensity) at 0.8 V vs RHE of high and low-energy peaks of double peaked reflectivity spectra in ACR regions of sample. Reflectivity change is normalised to ΔR at a 0 degrees polarisation angle for low energy peak. Error

bars are not shown but are approximately 15% of the values based on the signal-to-noise-ratios in the experiment.

Variations in reported haematite band-edge: In the literature the band-edge of haematite varies between 2.1 and 2.2 eV (560 to 590 nm), depending on the exact film morphology. For example, extreme nanostructure in the films can blue-shift the band-edge, $Sn^{(4+)}$ and C doping tends to red-shift the band edge[22], specific facets will also have an influence on the band-edge shifting it upwards of 0.2 eV[23] and other local features such as strain and electric field will also shift the band-edge. Given the limited energetic (2-4 nm) and spatial resolution (1 µm) in many of our techniques including the reflectivity measurements (which we believe cannot themselves interpret these small shifts) we do not to interpret them.

In summary whilst we cannot unequivocally rule out all possible origins of this second high-energy peak, our extensive new experiments and literature survey suggest it arises from a state related to band-edge Fe *d-d* transitions, much like the well-established low energy peak at 2.1 eV. Generally, we remark that while for a metallic electrode the energetics may depend on the bias, in the defective oxide electrodes we are working with, the morphology, as we have shown, can also plat a large role, i.e., bias does not necessarily solely determine energetic filling/ordering.

Supplementary Note 7: XANES and speciation study of hematite photoanodes

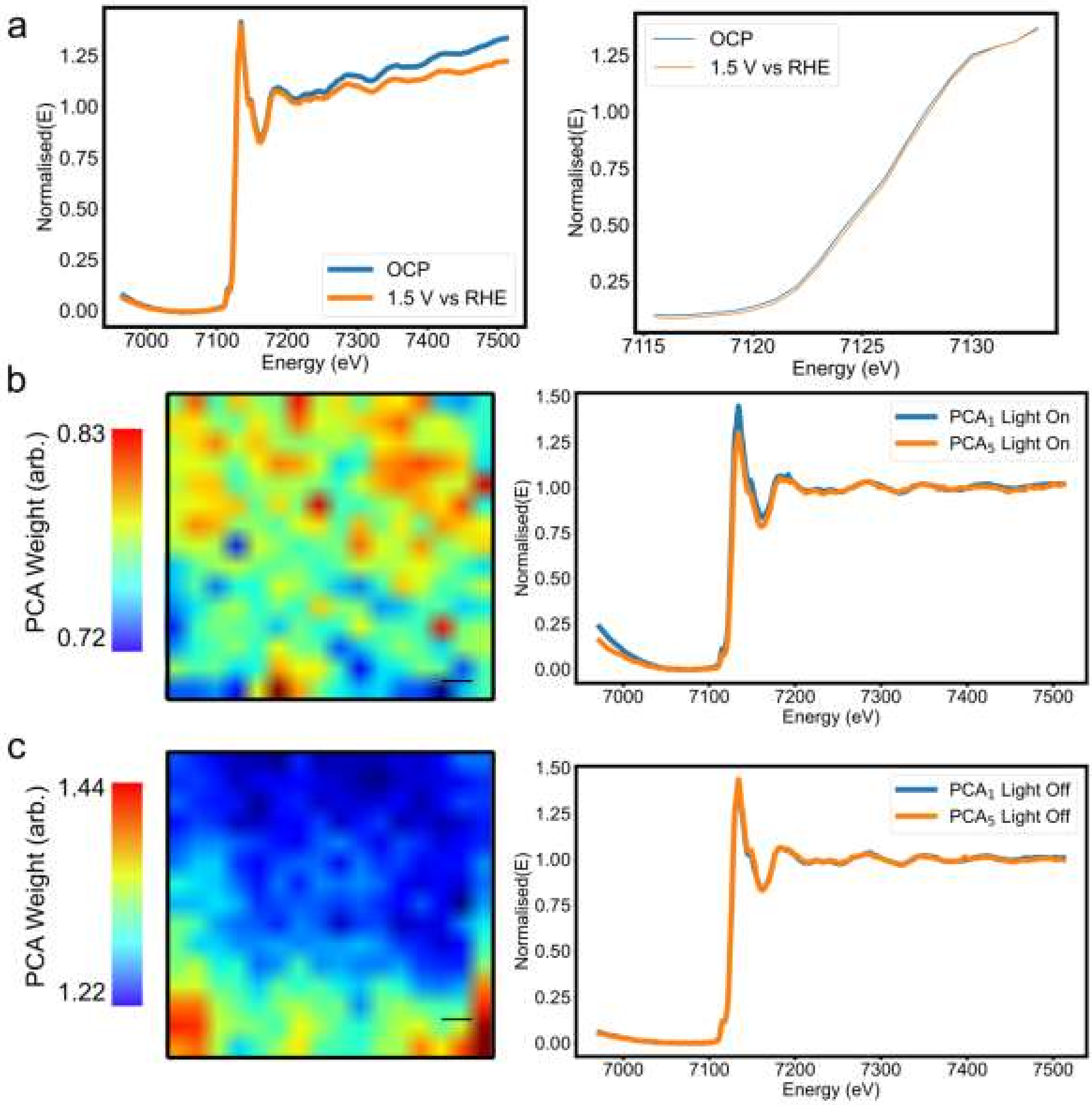

Supplementary Figure 13: a. XANES spectra of the hematite (α-Fe_2O_3) photoanode at open circuit potential (*i.e,* no applied potential, blue trace) and applying 1.5 V vs RHE (orange trace). The right shows a zooming into the band edge. Mapping and XANES spectra of undoped α-Fe_2O_3 photoanodes under dark (**b**) and light (**c**) at 1.5 V vs RHE. The maps correspond to an energy 7125 eV of with the XANES spectra of the different calculated clusters in dark and under illumination conditions shown in **b** and **c**, respectively.

Supplementary Figure 13a shows point XANES spectra of the hematite photoanode under open circuit potential and 1.5 V vs RHE, respectively, as control spectra for the spatially resolved XANES maps. The Fe K-edge XANES spectra exhibits a shift of~0.1 eV towards higher energies, i.e., higher oxidation state of the Fe, upon increasing the applied potential from OCP to 1.5 V vs. RHE as shown by the right panel of **Supplementary Figure 13a**. Given that at 1.5V under dark conditions the onset potential for photocurrent has not been reached, a shift in the absorption edge is expected due to the ionisation/oxidation mostly of the Fe atoms related to oxygen vacancies. The XANES were analysed using the MANTIS software (Multivariate analysis tool for spectro-microscopy, full description can be

found in reference Lerotic *et al.*[24]). Given that XANES data are collected in the spatial dimension (through pixels in *x* and *y*) with a spectrum per pixel, a cluster analysis is used to group pixels with similar eigenspectral after a principal component analysis (PCA) is used to extract a reduced set of such eigenspectra (**Figure 5b** and **c**).

Supplementary Note 8: Raw Raman spectra in 'dark' and 'light' conditions with bias

Raman measurements (off-resonant excitation 785 nm) have been performed under illumination. The illumination is from a white light with a 550 nm short-pass filter to prevent the illumination light interfering with the Raman signal (400-550 nm illumination). The whole area of electrode is illuminated simultaneously, and electrodes are pre-soaked with light for 50 mins before illumination to match the timescales of XANES measurements.

For the ACR/CR regions considered in **Figure 4** of the main text there is limited evolution of the Raman spectra under light. However, interestingly for the FL regions the intensity ratio of the 293/441 cm^{-1} and 611/660 cm^{-1} Raman peaks is (reversibly) altered. The ratio of these modes has been previously found to correlate positively with the photocurrent density at 1.23 V vs RHE, the location of intraband trap states, band gap and flat band potential. In particular, the magnitude of I_{293}/I_{411} is suggested to correlate with the properties of oxygen vacancies, whereas the magnitude of I_{611}/I_{660} the level of structural disorder and population of intraband states[25,26].

For the CR and ACR regions there is limited change in I_{293}/I_{411} and I_{611}/I_{660} in the light and dark (**Supplementary Figure 15**). However, interestingly for the FL region there is a systematic increase in I_{293}/I_{411} under illumination. For the I_{611}/I_{660} ratio there is a less clear change between dark and light conditions in FL but somewhat of a drop in the ratio particularly as the potential is raised. It is challenging to fully understand this behaviour but given the link between the 293/441 cm^{-1} mode ratio and oxide vacancies our results suggest in FL regions under illumination and bias there may be some changes in the vacancy population i.e., the vacancies and structural disorder are mobile. The mobility of OVs/disorder in α-Fe_2O_3 has not been measured to the best of our knowledge. However, in other oxide materials such as $SrTiO_3$ and TiO_2 the activation barrier height for oxide vacancy diffusion has been measured to be between 0.2 and 1.6 eV [27,28]. Interestingly, in these materials light is known to drive vacancy migration (when the material is held at bias) via Joule heating and the lowering of potential barriers associated with space charge zones that are depleted in charge carriers. Our observations suggest in 'native' thin films of haematite this may also be weakly possible, however further measurements e.g., the measurement of oxide vacancy barrier heights using STM or diffusion coefficients, using impedance methods, is needed. Finally, we note the microscopic lengthscales over which features like oxygen vacancies vary during the photoelectrochemical OER underscores the importance of moving of beyond bulk measurements, where such features are likely washed out and hence have been hitherto undetected. We also emphasise that given the limited quantification provided by Raman on features like OVs and general disorder, we cannot be sure that it is exactly OVs that are changing under light soaking or whether the changes we observe arise from other material properties (see discussion below).

For the T1/T2/T3 regions systematic changes in Raman peak ratios are harder to detect (**Supplementary Figure 17**). For I_{293}/I_{411} in T2/T3 there is very little change with and without light. Whereas for the T1 region which most closely resembles the native film (FL), there does indeed appear to be a change in I_{293}/I_{411} with/without light. For the I_{611}/I_{660} the changes with illumination are less clear, however this intensity ratio drops both in T3 and somewhat in T1 under light soaking and bias. These results broadly support the hypothesis that there is structural rearrangement in regions of the native film under bias and illumination. The exact nature of this remains to be further investigated.

We note that the above results are consistent with our μ-XANES measurements in **Figure 4** where structural changes were observed in the native film under bias and illumination. However, as we state throughout our work care has to be taken when comparing across techniques. For example, although the FL/T3 regions tend to show the greatest differences in our bias dependent Raman when illumination is added, they show a relatively small change in bias dependent reflectivity. However, this latter

measurement is effectively done in the dark, i.e., it is not measuring the effect of illumination on the OVs simply their population and how it responds to bias.

Finally, the intensity and width of the of E_g symmetry Raman modes of haematite have been shown to be related to OVs. However, as we highlight in the main these modes also report on the Fe-O bond stretch and O-O bond stretches and more generally static and dynamic disorder, that may or may not be related to OVs[29,30]. This contrasts with our differential reflectivity measurements which have been shown to be robustly linked exclusively to the OV concentration[31]. Hence, we use Raman to characterise more general disorder (which OVs fall under) and impurity phases such as FeOOH and reserve our reflectivity measurements for quantifying OV population specifically. Indeed, even though we can use Raman mode ratios to somewhat normalise for effects of sample thickness or laser penetration, in such inhomogeneous samples Raman is only semi-quantitative measure i.e., changes in Raman intensity/ratios reflect changes in the local structure (including OVs) but do not necessarily say if there is more or less OVs simply from their magnitude[25,26].

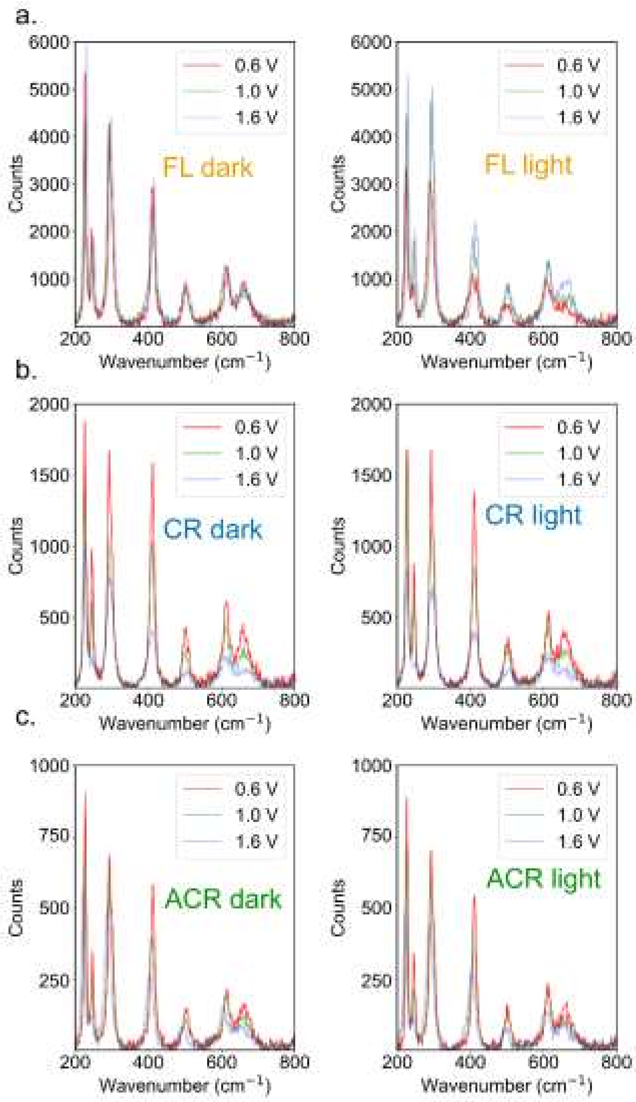

Supplementary Figure 14: a-c. Raw Raman spectra from FL (**a**), CR (**b**) and ACR (**c**) regions described in the main text, under bias dark/light (Xenon white light >500 μm^2 illumination area). For the CR region the spectra have been averaged over 4 pixels to improve the signal-to-noise ratio and in the ACR region over 9 pixels. The spectra are truncated at 800 cm^{-1} such that the high-intensity peak at 1320 cm^{-1} does not obscure examination.

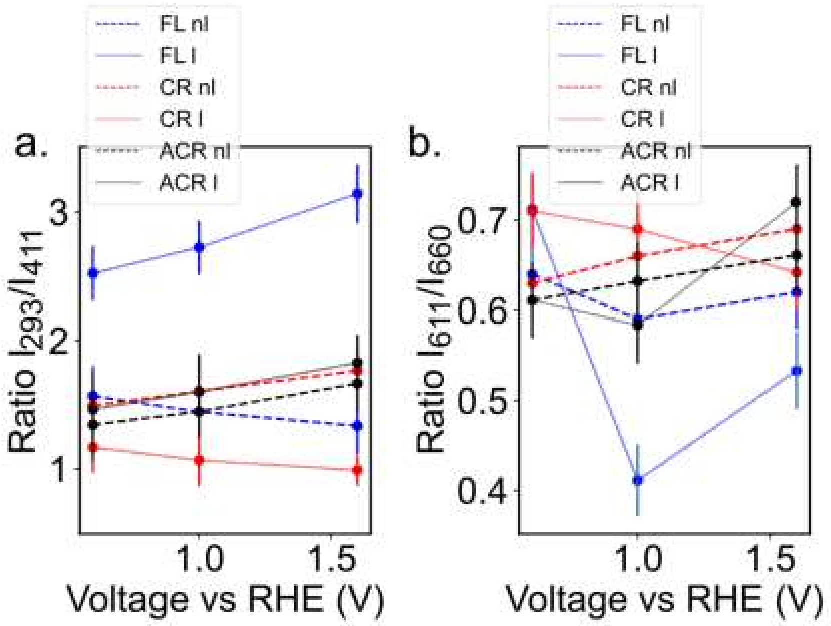

Supplementary Figure 15: a-b. Ratio of Raman mode intensities I_{293}/I_{411} (**a**) and I_{611}/I_{660} (**b**) for FL, CR ACR regions with (l) and without light (nl) as a function of bias. Error bars estimated by propagating uncertainty of peak intensity from experimental signal-to-noise-ratio.

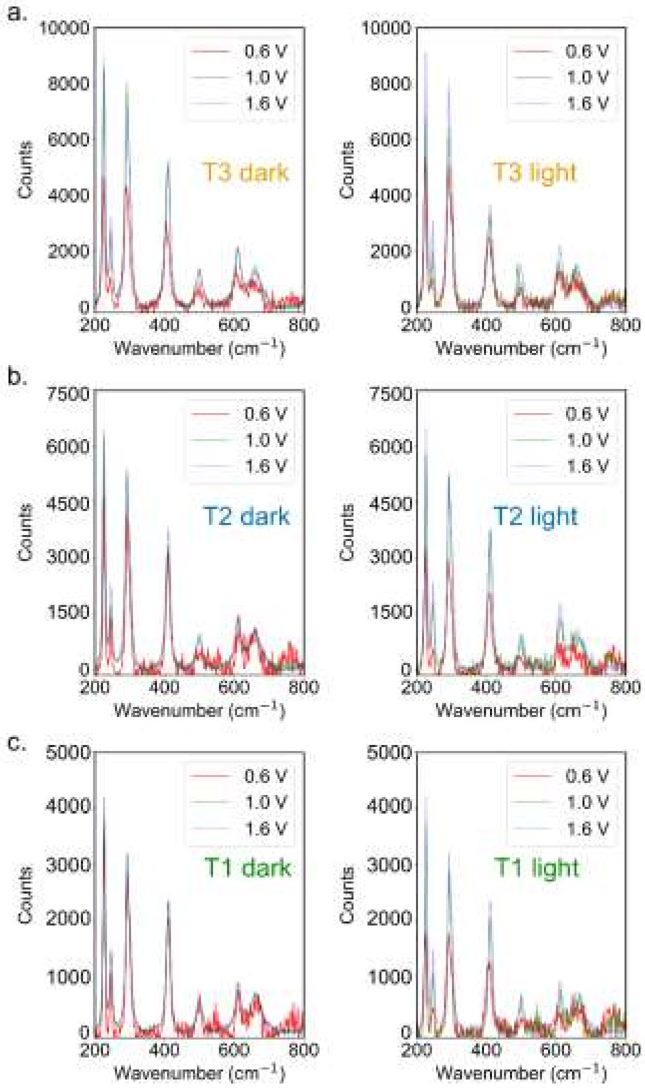

Supplementary Figure 16: a-c. Raw Raman spectra from T3 (**a**), T2 (**b**) and T1 (**c**) regions described in the main text, under bias dark/light (Xenon white light >500 μm^2 illumination area). For the CR region the spectra have been averaged over 4 pixels to improve the signal-to-noise ratio and in the ACR region over 9 pixels. The spectra are truncated at 800 cm^{-1} such that the high-intensity peak at 1320 cm^{-1} does not obscure examination.

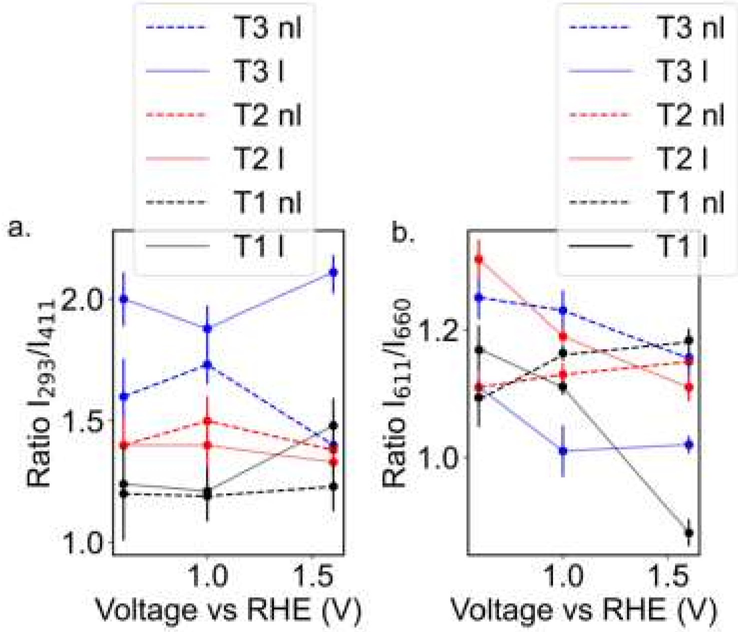

Supplementary Figure 17: a-b. Ratio of Raman mode intensities I_{293}/I_{411} (**a**) and I_{611}/I_{660} (**b**) for T3, T2 and T1 regions with (l) and without light (nl) as a function of bias. Error bars estimated by propagating uncertainty of peak intensity from experimental signal-to-noise-ratio.

Supplementary Note 9: Structural changes under light and bias

The region examined by μ-XANES is effectively a homogeneous region of the α-Fe_2O_3 sample. We observe under bias and light soaking there is a clear structural rearrangement of disorder, and potentially vacancies, in the films in this region.

To understand if this behaviour can also be observed in the microscale electrochemical performance of samples, we have performed potentiostatic mapping after 5, 20, 60 and 120 min of continuous light soaking (400-900 nm) of the entire electrode under 1.5 V vs RHE bias (**Supplementary Figure 18**). After 5, 20 min no clear patterns/segregation emerges in the maps of current density at 1.5 V vs RHE. However, after 60 mins some small local segregation in the photocurrent density emerges in the maps. After 120 min this appear to be still present but with little change. After ramping the films back to the open circuit potential, and leaving them in the dark for 10 mins, the maps of current density at 1.5 V vs RHE once again appear to be homogeneous. These results support the idea of some changes in structure/disorder from extended light exposure which can impact the overall local performance in the film. However, it is unclear exactly the mechanism for such reconstruction i.e., is there underlying nanostructure in the film that enables this?

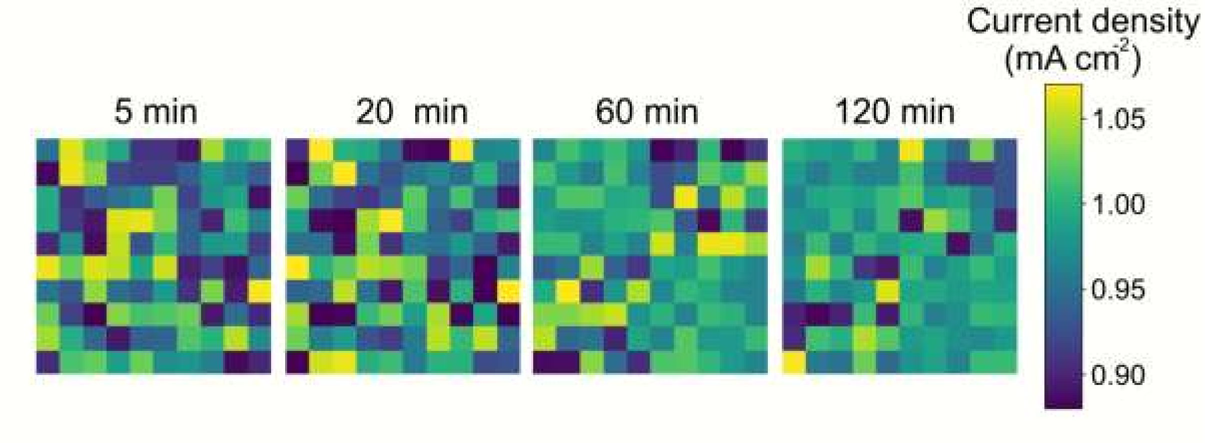

Supplementary Figure 18: Maps of photocurrent at 1.5 V vs RHE across homogeneous region of hematite film shown in **Supplementary Figure 20**. Each pixel is 1 μm. The time indicates the period for which the electrode is illuminated with a Xe white light 500 μm^2> illumination area. (Same as **Figure 4f** but with values plotted as pixels).

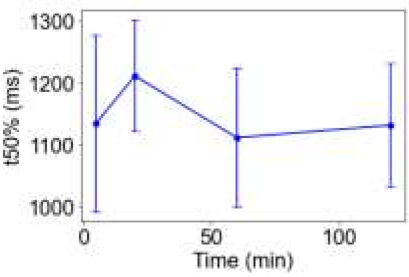

Supplementary Figure 19: $t_{50\%}$ values at 1.6 V vs RHE across a selection of pixels indicated with red stars in **Supplementary Figure 20**. Error bars derived from standard deviation across values.

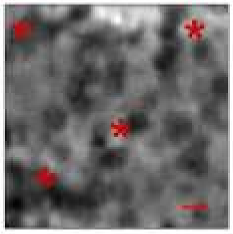

Supplementary Figure 20: Brightfield optical image of haematite thin-film. Scale bar is 1 µm. Red asterisks show locations where transient kinetics are measured.

Finally, we did also measure the $t_{50\%}$ values and reflection spectra after 5, 20, 60 and 120 min of continuous light-soaking (as above) at 1.5 V_{RHE} (**Supplementary Figure 19** and **20**). However, we found no statistically significant changes in these measurements over time. This may be related the fact that the pathways are not necessarily affecting the recombination dynamics or the reduced sensitivity of the transient reflection microscopy as compared to XANES. Indeed, given the uncertainty on $t_{50\%}$ values is ~10%, which is similar to the spatial variation in photocurrents, this may be the main reason why we cannot observe any differences. Regardless, further experiments particularly with regards to quantifying oxygen vacancy mobility in haematite, are necessary.

Supplementary Note 10: Sn concentration distribution across films and oxygen concentration of T1, T2 and T3

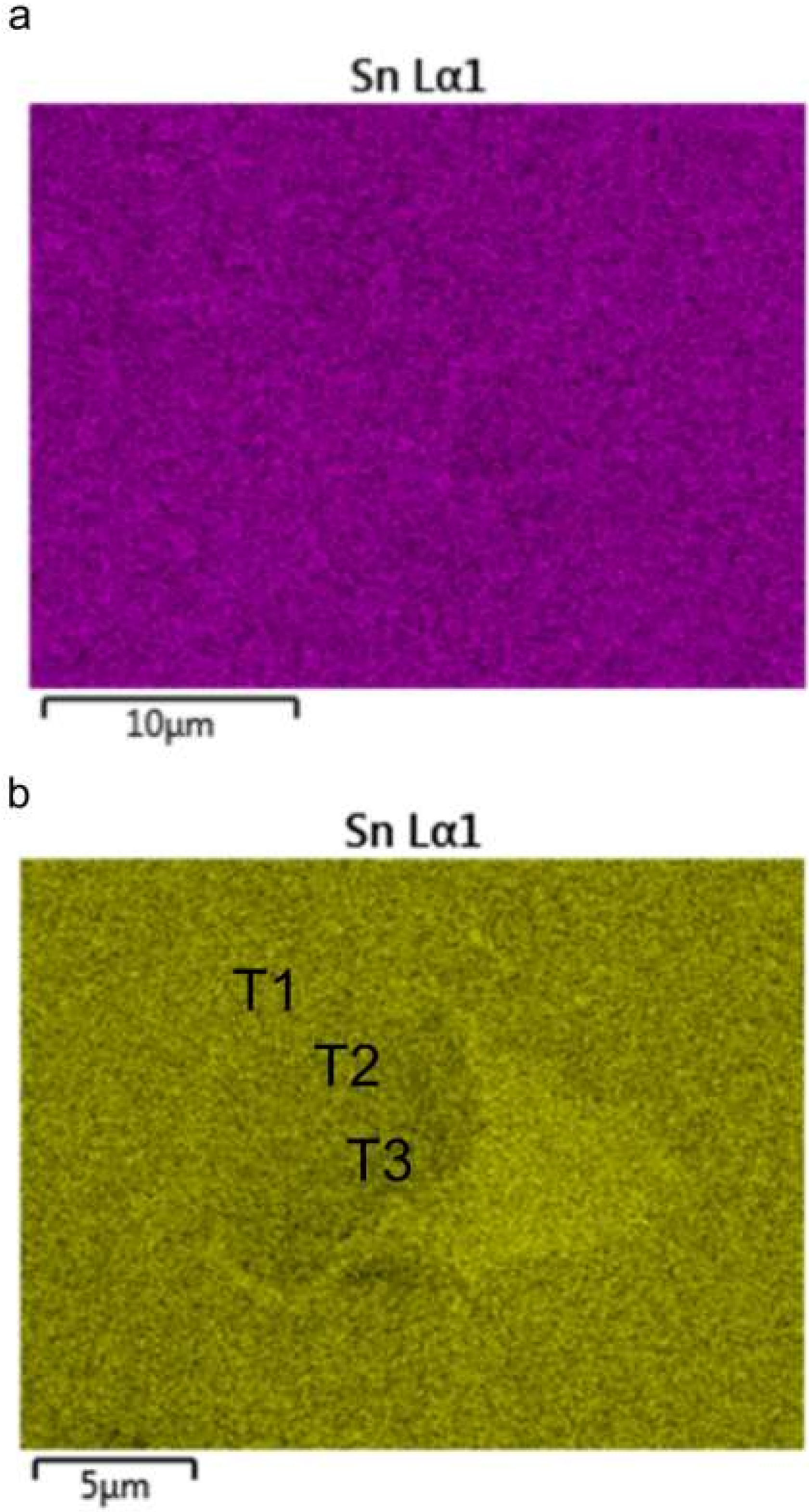

Supplementary Figure 21: a. EDX chemical maps showing Sn content. In the region mapped in **Figure 3** and **4** of the main text there is a uniform Sn concentration between CR, ACR and FL regions.

Hence improved photocurrent cannot be ascribed to diffusion of Sn into the film which would facilitate more facile charge extraction[32]. **b.** In the region mapped in **Figure 5** and **6** of the main text T1 shows a slightly higher Sn content as compared to T2 and T3.

The O content slightly drops on-going from T3 to T1 (as shown in **Supplementary Figure 22)**. This is consistent with the reflectivity measurements that suggest T1 also has a larger number of oxygen vacancies. This result is also somewhat in agreement with our assertion that T3 is similar to FL like regions. However, some care is needed as the oxygen content from EDX do not reflect purely oxygen vacancies.

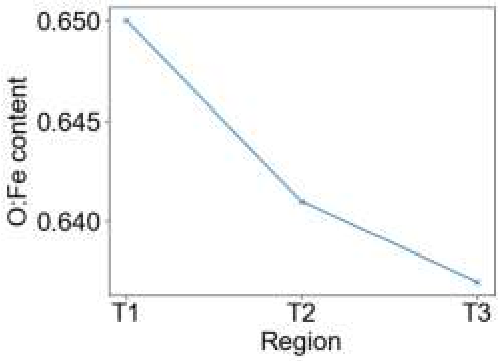

Supplementary Figure 22: O content for T1, T2 and T3 regions obtained from EDX. Error on values is just below 0.05.

Supplementary Note 11: Correcting for differences in absorbance between different regions of sample

At the wavelength we measure the photocurrent locally, α-Fe_2O_3 has an absorption coefficient of 4×10^4 cm^{-1} (this absorption coefficient is generally wavelength independent and is on the same order of magnitude for films synthesised using the routes in this work). Based on the Beer-Lamber law the light intensity will decay as shown in **Supplementary Figure 23** inside the sample. The axial extent of our objective is ~500 nm which means we must consider this decay in light intensity when calculating any quantities based on light absorption on our samples (namely the photocurrent and reflectivity). This ~500 nm axial extent prevents us from measuring the curve in **Supplementary Figure 23** experimentally for our samples, but it has been reported also by other groups[33]. To normalise for this thickness variation between the T1, T2 and T3 regions considered in **Figure 5**, we can multiply the photocurrent measured at each voltage and spatial position by a pre-factor $exp^{(\Delta z\times\alpha)}$ where Δz is the difference in film thickness compared to the nominal film thickness of 300 nm and α the attenuation coefficient. In this way any trends in photocurrent we compare are free from thickness/light absorption effects. Importantly, both with and without this correction our trends in the photocurrent and onset potential remain identical.

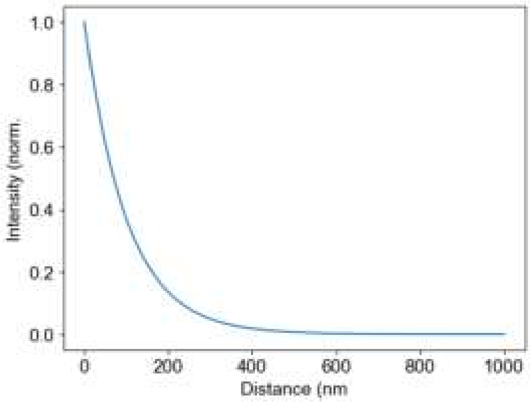

Supplementary Figure 23: Exponential decay of α-Fe_2O_3 absorption based on Beer-Lambert law.

With regards to the reflectivity measurements deriving any correction based on thickness is more challenging. The predominant reflection will be from the FTO/haematite interface based on refractive index differences and our objective focus position. While there will be some evanescent optical field into the material, the surface reflectivity we are measuring should in our view be independent of thickness. Indeed, we note that the magnitude of the ΔR/R signal follows a trend of T3>T2>T1 despite T3 being the thinnest region. Based on the above rationale we do not apply the above correction to the reflectivity spectra (or ΔR/R signals) but note that our trends remain the same, as shown by **Supplementary Figure 24**, if scaling was applied. As we do not draw a quantitative value on the oxygen vacancy concentration the exact value is of little importance for the conclusions set out in this work.

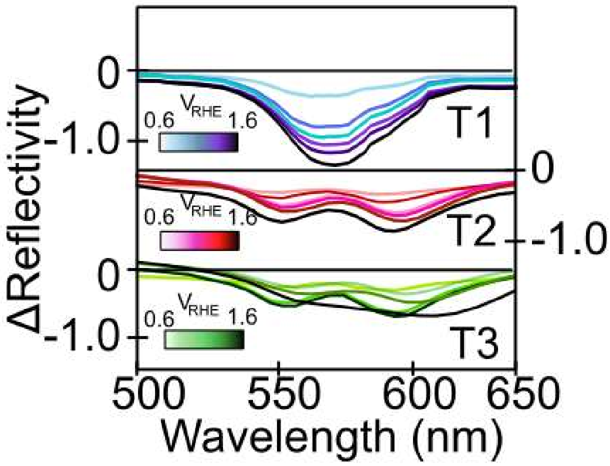

Supplementary Figure 24: Differential spectral reflectivity (with respect to reflection spectrum at 0.5 V_{RHE}) as a function of bias from T1, T2 and T3, after correcting for all regions having a nominal thickness of 300 nm. The trends in the magnitude and shape of the reflectivity spectra remain the same after scaling.

Supplementary Note 12: Magnitude of ΔR/R signal depending on spatial location for hematite regions with different thicknesses

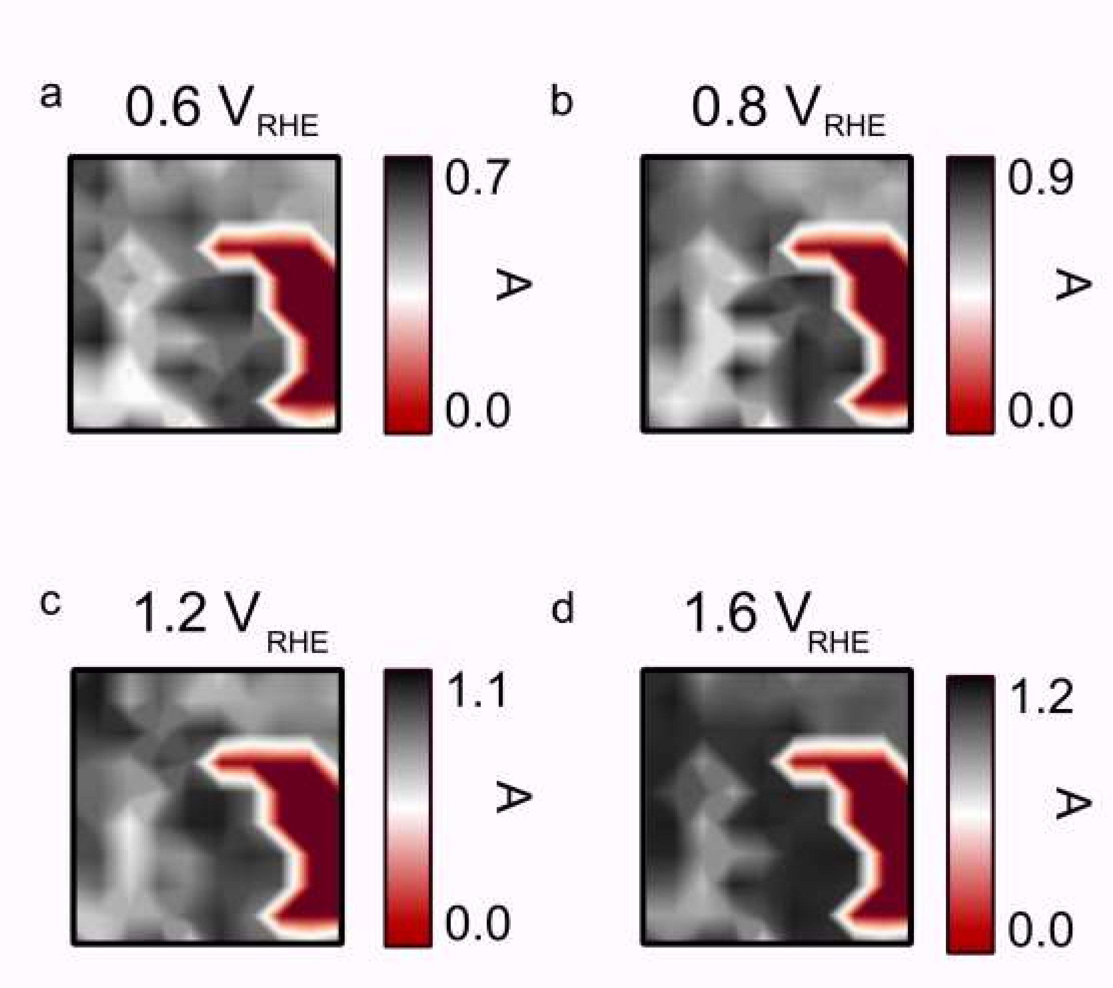

Supplementary Figure 25: a-d. Spatial maps of magnitude of ΔR/R kinetic (A) as a function of spatial location at potentials of 0.6 to 1.6 V_{RHE}. The magnitude of the ΔR/R signal also is ~1.4 to 1.7 times larger in T2/T3 as compared to T1 depending on the potential and location.

Supplementary Note 13: The effects of carbon impurities

As well as microstructural/point defects metal oxide catalysts also display chemical impurities. Microscopic inspection coupled with SEM and EDX analysis identifies 10 to 25 µm wide and 2 to 3 µm high residues on the surface of the hematite films as indicated partially by the white box in **Supplementary Figure 26a**. Mapping the photocurrent density across a 10 × 10 um region containing both thin film Fe_2O_3 (FL2) and the residue region (CCI) shows a stark increase in the potential required for the onset of photocurrent generation from these latter regions; 1.15 V_{RHE} for CCI *versus* 0.85 V_{RHE} for FL2 (**Supplementary Figure 26b**). Furthermore, the photocurrent density at 1.6 V_{RHE}, i.e., where mostly water oxidation occurs, is higher in FL2 compared to CCI regions. This suggests that there is a higher population of generated holes in this region which is in-keeping with the higher optical density at 550 nm (hematite band edge), for FL2 (see **Supplementary Figure 27**), i.e., more absorption in FL2. Indeed, while we do not correct for the absorption/attenuation differences in our photocurrent measurements of CCI and FL2 we note that this difference in absorption would only further accunate the trend.

Elemental analysis shown in **Supplementary Figure 26c** reveals that the CCI residues are still hematite based but contain a large proportion of carbon, potentially left from the synthesis process[34]. The carbon manifests itself as a strong peak at 0.28 eV in the EDX spectrum in **Supplementary Figure 28**. Although the Fe content in CCI and FL2 is the same (false colour images in **Supplementary Figure 26c**), CCI regions are oxygen deficient; O:Fe ratio in CCI regions is 0.52 *versus* 0.64 in FL2.

To further understand the role played by carbon impurities in reducing the activity we trace the hole lifetime over the same region in **Supplementary Figure 29**. Interestingly, in CCI regions $t_{50\%}$ is slightly longer at all potentials than in FL2, e.g., $t_{50\%}$ ~1450 ms at 1.6 V_{RHE} in CCI, whereas in FL2 at the same potential $t_{50\%}$ ~ 1210 ms. These observations are slightly at odds with the higher photocurrent density onset in CCI regions which would suggest that BER is more significant and water oxidation requires a higher potential in these regions. However, previous reports have shown that when hematite is templated onto carbon nanorods[35] and carbon nanonets[36], the hole lifetime can be enhanced due to an increased mobility of holes and reduced recombination. We postulate a similar effect occurs here with the carbon impurities (which in our case are much thicker) acting to increase the hole lifetime offsetting BER pathways and marginally increasing the overall decay time. If a junction is formed between the hematite surface and the carbon impurities additional band bending may further reduce hole recombination. The low photocurrent efficiency, despite longer-lived holes, then likely arises from the small hole population as a result of the poor absorption in CCI compared to FL2 and the fact that the (thick) carbon layer likely inhibits transfer to the electrolyte. This is corroborated by the lower ΔR/R signal magnitude for kinetics in this region as shown in **Supplementary Figure 29b**.

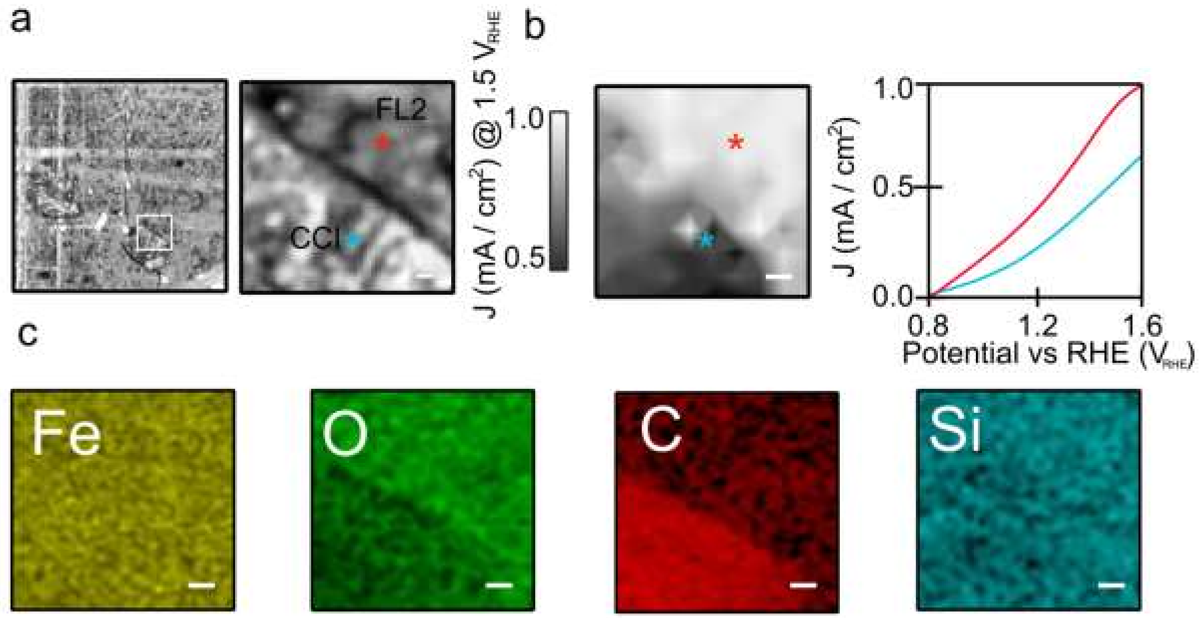

Supplementary Figure 26: a. Optical image of hematite thin film containing carbon impurity regions. White box marks shows zoomed in 10 µm^2 region examined. Blue and red asterisks mark carbon impurity (CCI) and film regions (FL2). **b.** Photocurrent density map at 1.5 V_{RHE} across region highlighted in a. **c.** SEM/EDX analysis of carbon impurity containing (CCI) and thin film regions of sample (FL2). False colour EDX maps show the presence of carbon in the CCI regions and the existence of oxygen deficiencies. Image brightness qualitatively indicates percentage abundance. All scale bars are 1 µm.

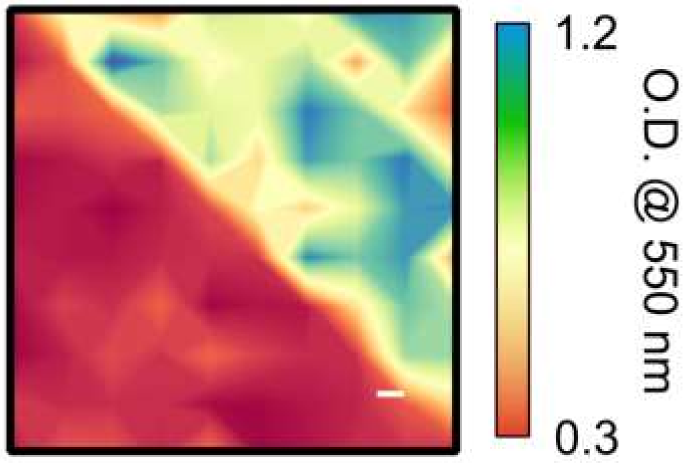

Supplementary Figure 27: Optical density (OD) at 550 nm of region studied in **Supplementary Figure 26**. Scale bar is 1 µm.

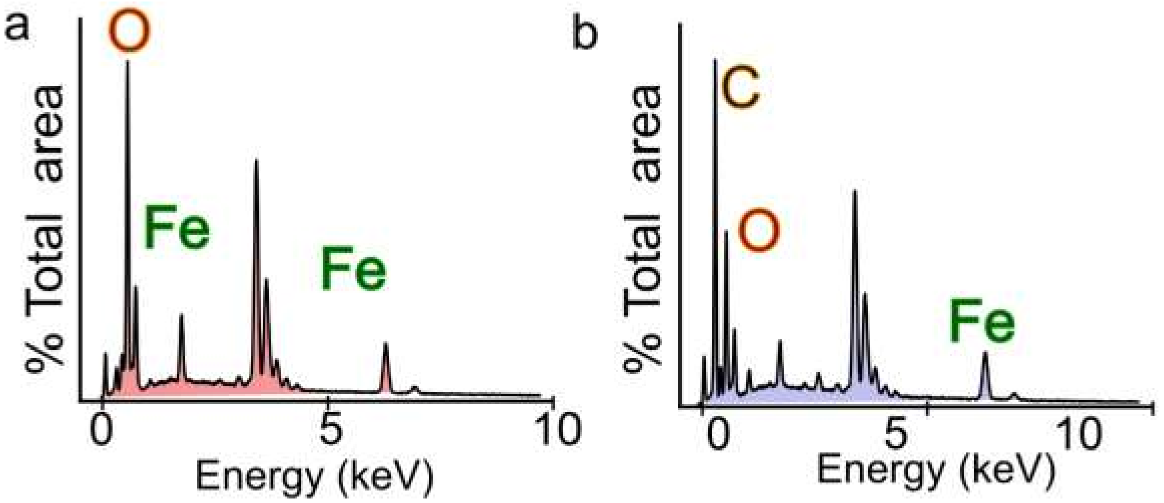

Supplementary Figure 28: a. EDX spectrum of thin film regions (FL2) of α-Fe_2O_3 electrode. **b.** EDX spectrum of carbon impurity containing (CCI) regions of sample. A strong peak at 0.277 eV confirms the presence of carbon in the spectra of CCI regions, the Fe and O EDX peaks are additionally marked.

In **Supplementary Figure 29c** we plot the differential spectral reflectivity from FL2 and CCI with increasing bias. A characteristic negative peak is observed at ~580 nm in both, but this is of strikingly low intensity in CCI and does not significantly evolve with bias. This not only indicates that there is likely a large degree of absorption from the carbon but also possibly a very low [OV] here, i.e., vacancies do not play a large role in facilitating charge transport even after accounting for the low amounts of hematite present. Raman mapping of the sample regions shows the intensity and width of the $A^1{}_g$, E_g and FeOOH bands is lower throughout the CCI regions as compared to compared to FL2 (**Supplementary Figure 30**). The position of the $A^1{}_g$ and E_g modes is shifted to lower frequencies in the CCI regions as compared to the FL2 regions, whereas for the E_u mode this trend is reversed. Surprisingly, no new Raman bands corresponding to carbon impurities can be observed. This suggests that the carbon impurities do not interact *via* a crystalline bonding network with the Fe_2O_3 lattice and are instead amorphously connected, i.e., the impurities do not significantly disrupt the crystal structure. In the CCI region, bottom right panel **Supplementary Figure 29d**, there is almost no change in the Raman spectrum under bias. This stark contrast to the behaviour in the FL2 regions (or ACR/CR and T1T2/T3 in the main text) suggests that there is little change in bond lengths or vacancy environment/population during the water oxidation in this region.

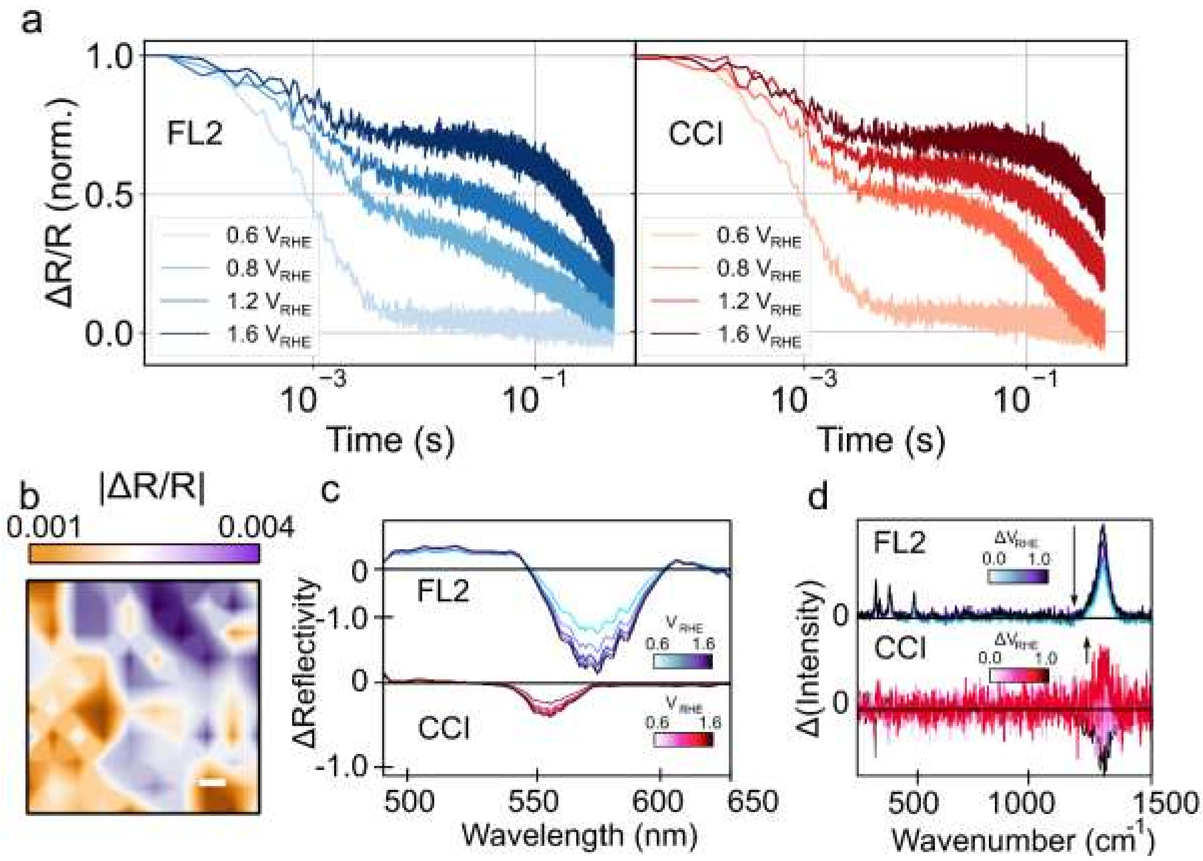

Supplementary Figure 29: a. Kinetics of hole decay from FL2 and CCI regions as a function of bias. **b.** Absolute magnitude of |ΔR/R| signal as a function of spatial position over region shown in **Supplementary Figure 26a**. Scale bar is 1 µm. **c.** Differential spectral reflectivity (with respect to reflection spectrum at 0.5 V_{RHE}) as a function of bias from CCI and FL2 regions. The former shows a significantly smaller change in reflectivity under bias. **d.** Differential Raman spectra (with respect to 0.5 V_{RHE}) from spatial positions marked in **Supplementary Figure 26a**.

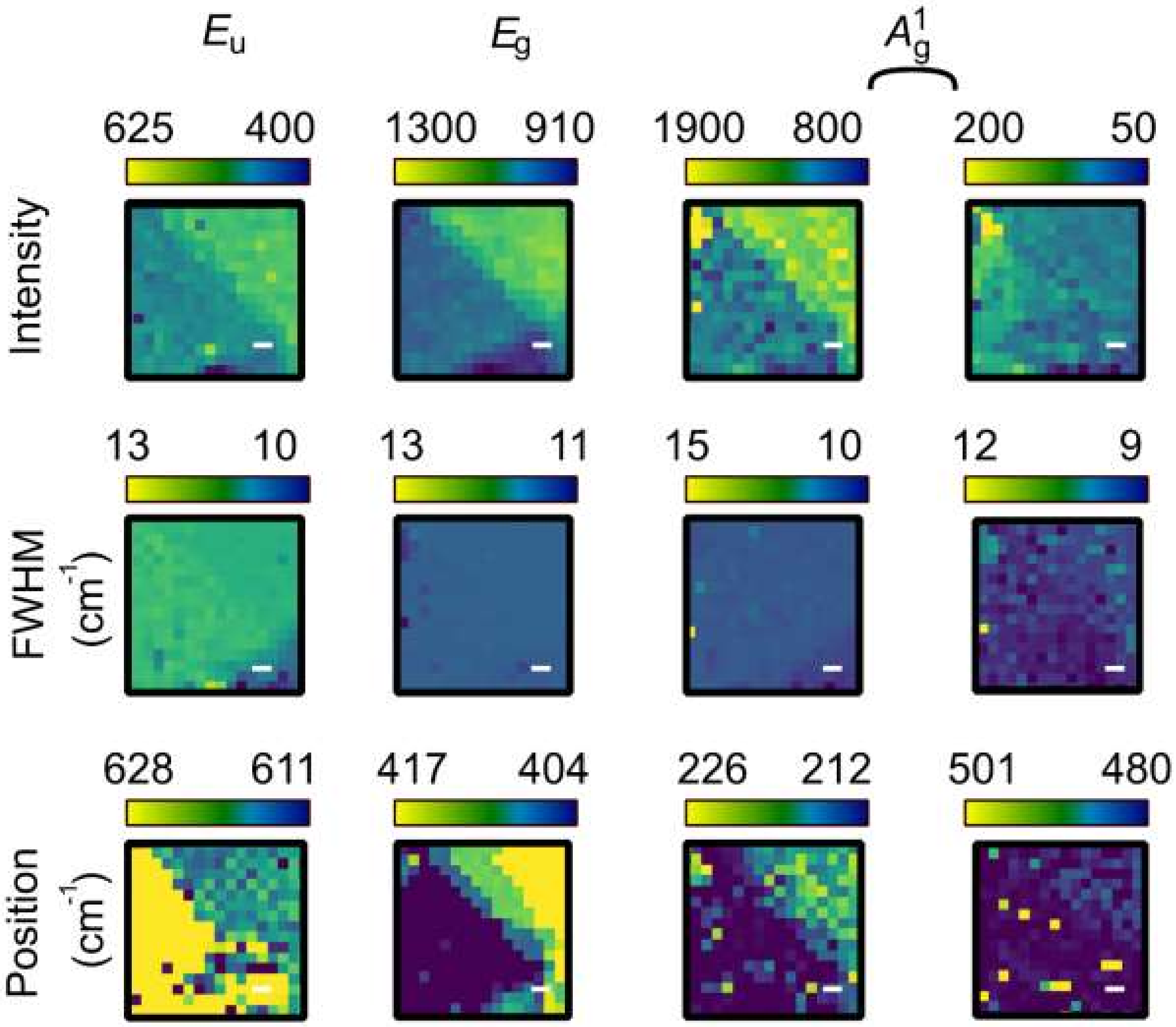

Supplementary Figure 30: Image of intensity, FWHM and position of indicated Raman modes from the CCI and FL2 regions indicated in **Supplementary Figure 26**.

Supplementary Note 14: Pump wavelength and fluence dependence of transient microscopy results

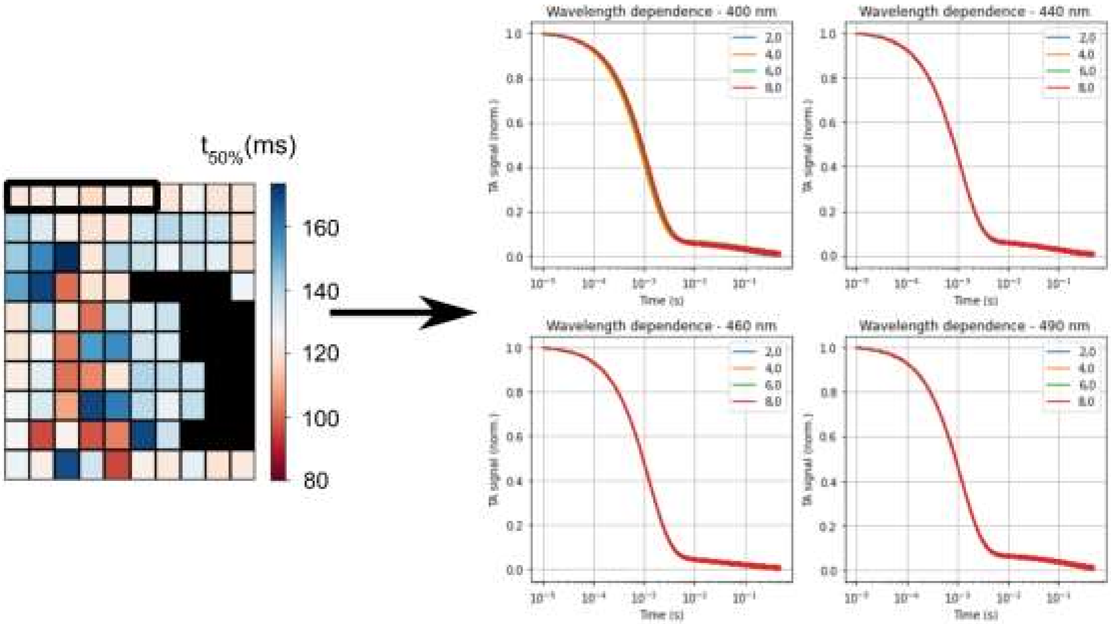

Supplementary Figure 31: Pump wavelength dependence of spatial dynamics of thinned sample regions (**Figure 5** and **6** of main text) at 0.6 V_{RHE}. Spatial map of $t_{50\%}$ values (left) and corresponding (smoothed) kinetics (right) for various pump pulse wavelengths at alternate pixels indicated in the black box. No significant variation is observed based on the pump wavelength. Blacked squares are substrate. Each pixel is 1 µm. The fitting error on $t_{50\%}$ is ~10%.

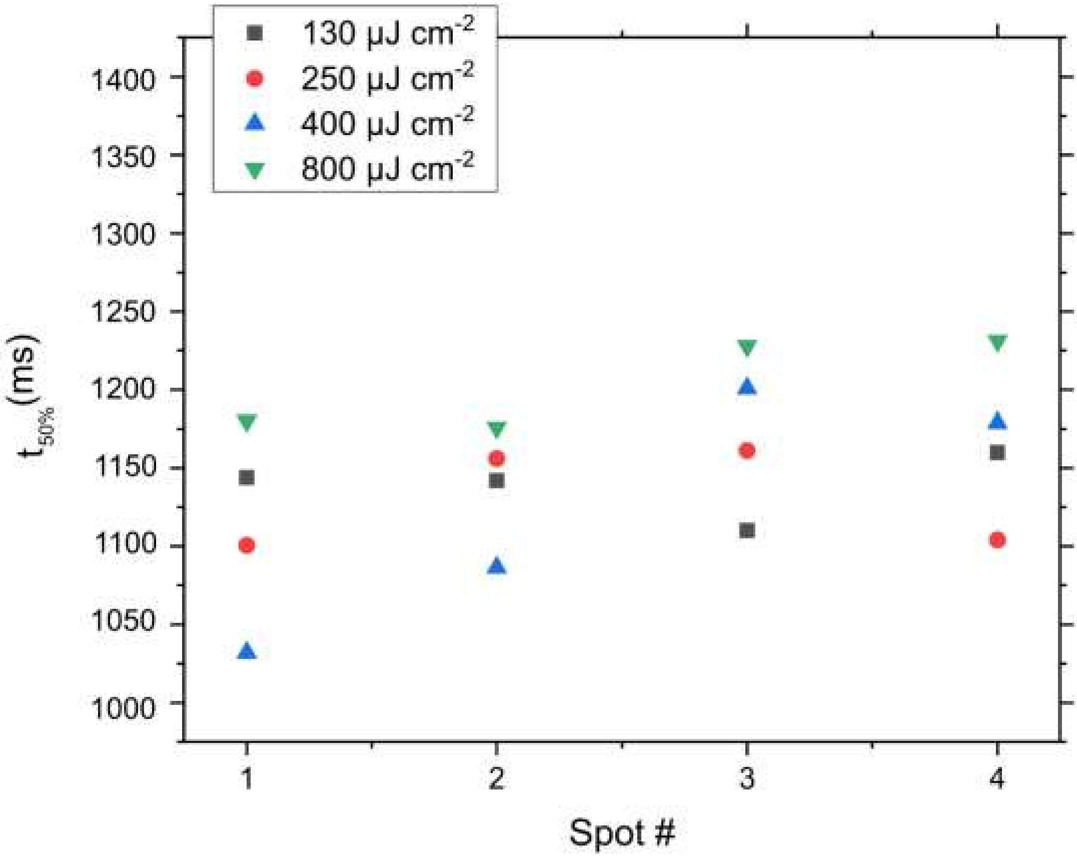

Supplementary Figure 32: Pump fluence dependence of $t_{50\%}$ dynamics (at 1.6 V_{RHE}) as a function of spatial position for pixel 2,0 as shown in **Supplementary Figure 31**. The differences in $t_{50\%}$ are no greater than ~150 ms between fluences which is larger than the vairiation observed between different spatial regions of the sample (~300 to 500 ms). This limited fluence dependence is consistent with previous reports[37]. The fitting error on $t_{50\%}$ is ~10%.

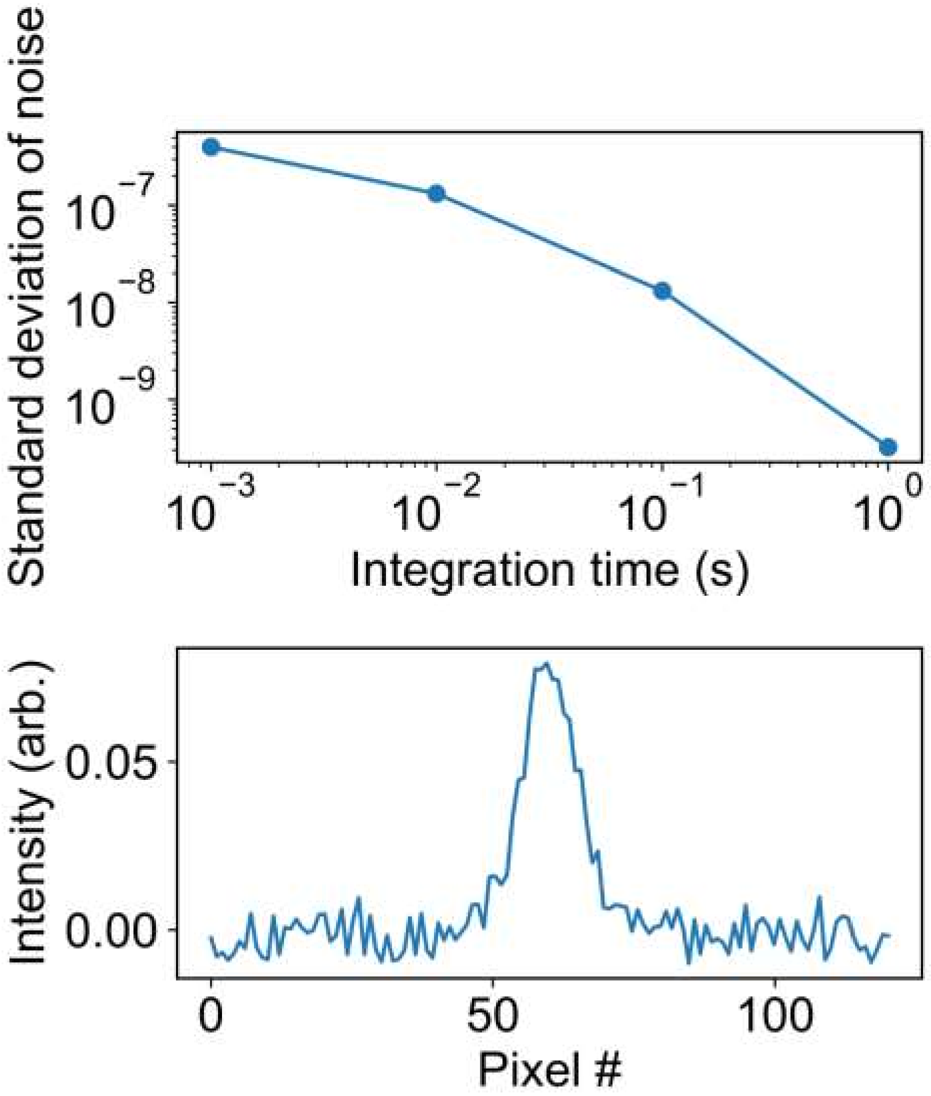

Supplementary Figure 33: (Top) Shot noise characterisation curve of pump-probe microscope. **(Bottom)** Each pixel on the camera is 3.1 microns in size. With the effective magnification of our imaging system 140× we estimate a pump spot FWHM of ~500 nm.

Supplementary Reference

(1) Zandi, O.; Hamann, T. W. Enhanced Water Splitting Efficiency through Selective Surface State Removal. *J. Phys. Chem. Lett.* **2014**, *5* (9), 1522–1526.

(2) Mesa, C. A.; Steier, L.; Moss, B.; Francà, L.; Thorne, J. E.; Grä, M.; Durrant, J. R. Impact of the Synthesis Route on the Water Oxidation Kinetics of Hematite Photoanodes. *J. Phys. Chem. Lett* **2020**, *11*, 23.

(3) Kay, A.; Cesar, I.; Grätzel, M. New Benchmark for Water Photooxidation by Nanostructured α-Fe 2O3 Films. *J. Am. Chem. Soc.* **2006**, *128* (49), 15714–15721.

(4) Hodes, G. Photoelectrochemical Cell Measurements: Getting the Basics Right. *J. Phys. Chem. Lett.* **2012**, *3* (9), 1208–1213.

(5) Misho, R. H.; Murad, W. A. Band Gap Measurements in Thin Films of Hematite Fe2O3, Pyrite FeS2 and Troilite FeS Prepared by Chemical Spray Pyrolysis. *Sol. Energy Mater. Sol. Cells* **1992**, *27* (4), 335–345.

(6) Klahr, B.; Gimenez, S.; Fabregat-Santiago, F.; Hamann, T.; Bisquert, J. Water Oxidation at Hematite Photoelectrodes: The Role of Surface States. *J. Am. Chem. Soc.* **2012**, *134* (9), 4294–4302.

(7) Li, Y.; Guijarro, N.; Zhang, X.; Prévot, M. S.; Jeanbourquin, X. A.; Sivula, K.; Chen, H.; Li, Y. Templating Sol-Gel Hematite Films with Sacrificial Copper Oxide: Enhancing Photoanode Performance with Nanostructure and Oxygen Vacancies. *ACS Appl. Mater. Interfaces* **2015**, *7* (31), 16999–17007.

(8) Wang, Z.; Mao, X.; Chen, P.; Xiao, M.; Monny, S. A.; Wang, S.; Konarova, M.; Du, A.; Wang, L. Understanding the Roles of Oxygen Vacancies in Hematite-Based Photoelectrochemical Processes. *Angew. Chemie Int. Ed.* **2019**, *58* (4), 1030–1034.

(9) Mesa, C. A.; Steier, L.; Moss, B.; Francàs, L.; Thorne, J. E.; Grätzel, M.; Durrant, J. R. Impact of the Synthesis Route on the Water Oxidation Kinetics of Hematite Photoanodes. *J. Phys. Chem. Lett.* **2020**, *11* (17), 7285–7290.

(10) Mesa, C. A.; Francàs, L.; Yang, K. R.; Garrido-Barros, P.; Pastor, E.; Ma, Y.; Kafizas, A.; Rosser, T. E.; Mayer, M. T.; Reisner, E.; et al. Multihole Water Oxidation Catalysis on Haematite Photoanodes Revealed by Operando Spectroelectrochemistry and DFT. *Nat. Chem.* **2019**, *12*, 82–89.

(11) Wood, S.; O'Connor, D.; Jones, C. W.; Claverley, J. D.; Blakesley, J. C.; Giusca, C.; Castro, F. A. Transient Photocurrent and Photovoltage Mapping for Characterisation of Defects in Organic Photovoltaics. *Sol. Energy Mater. Sol. Cells* **2017**, *161*, 89–95.

(12) Wu, J.; Yang, D.; Liang, J.; Werner, M.; Ostroumov, E.; Xiao, Y.; Watanabe, K.; Taniguchi, T.; Dadap, J. I.; Jones, D.; et al. Ultrafast Response of Spontaneous Photovoltaic Effect in 3R-MoS2-Based Heterostructures. *Sci. Adv.* **2022**, *8* (50).

(13) Klahr, B.; Hamann, T. Water Oxidation on Hematite Photoelectrodes: Insight into the Nature of Surface States through in Situ Spectroelectrochemistry. *J. Phys. Chem. C* **2014**, *118* (19), 10393–10399.

(14) Snir, N.; Toroker, M. C. The Operando Optical Spectrum of Hematite during Water Splitting through a GW-BSE Calculation. *J. Chem. Theory Comput.* **2020**, *16* (8), 4857–4864.

(15) Cummings, C. Y.; Marken, F.; Peter, L. M.; Upul Wijayantha, K. G.; Tahir, A. A. New Insights into Water Splitting at Mesoporous α-Fe 2O 3 Films: A Study by Modulated Transmittance and Impedance Spectroscopies. *J. Am. Chem. Soc.* **2012**, *134* (2), 1228–1234.

(16) Hinrichsen, T. F.; Chan, C. C. S.; Ma, C.; Paleček, D.; Gillett, A.; Chen, S.; Zou, X.; Zhang, G.; Yip, H. L.; Wong, K. S.; et al. Long-Lived and Disorder-Free Charge Transfer States Enable Endothermic Charge Separation in Efficient Non-Fullerene Organic Solar Cells. *Nat. Commun. 2020 111* **2020**, *11* (1), 1–10.

(17) Bublitz, G. U.; Boxer, S. G. Stark Spectroscopy: Applications in Chemistry, Biology, and Materials Science. *Annu. Rev. Phys. Chem.* **1997**, *48*, 213–242.

(18) Piccinin, S. The Band Structure and Optical Absorption of Hematite (α-Fe2O3): A First-Principles GW-BSE Study. *Phys. Chem. Chem. Phys.* **2019**, *21* (6), 2957–2967.

(19) Beermann, N.; Vayssieres, L.; Lindquist, S.-E.; Hagfeldt, A. Photoelectrochemical Studies of Oriented Nanorod Thin Films of Hematite. *J. Electrochem. Soc.* **2000**, *147* (7), 2456.

(20) Marusak, L. A.; Messier, R.; White, W. B. Optical Absorption Spectrum of Hematite, AFe2O3 near IR to UV. *J. Phys. Chem. Solids* **1980**, *41* (9), 981–984.

(21) Aziz, W. J.; Abid, M. A.; Kadhim, D. A.; Mejbel, M. K. Synthesis of Iron Oxide (β-Fe2o3) Nanoparticles from Iraqi Grapes Extract and Its Biomedical Application. *IOP Conf. Ser. Mater. Sci. Eng.* **2020**, *881* (1), 012099.

(22) Ahn, H. J.; Kment, S.; Naldoni, A.; Zbořil, R.; Schmuki, P. Band Gap and Morphology Engineering of Hematite Nanoflakes from an Ex Situ Sn Doping for Enhanced Photoelectrochemical Water Splitting. *ACS Omega* **2022**, *7* (39), 35109–35117.

(23) Xiang, Q.; Chen, G.; Lau, T. C. Effects of Morphology and Exposed Facets of α-Fe 2 O 3 Nanocrystals on Photocatalytic Water Oxidation. *RSC Adv.* **2015**, *5* (64), 52210–52216.

(24) Lerotic, M.; Mak, R.; Wirick, S.; Meirer, F.; Jacobsen, C. MANTiS: A Program for the Analysis of X-Ray Spectromicroscopy Data. *J. Synchrotron Radiat.* **2014**, *21* (5), 1206–1212.

(25) Liu, Y.; Smith, R. D. L. Differentiating Defects and Their Influence on Hematite Photoanodes Using X-Ray Absorption Spectroscopy and Raman Microscopy. *ACS Appl. Mater. Interfaces* **2022**, *14* (5), 6615–6624.

(26) Liu, Y.; Smith, R. D. L. Identifying Protons Trapped in Hematite Photoanodes through Structure–Property Analysis. *Chem. Sci.* **2020**, *11* (4), 1085–1096.

(27) Scheiber, P.; Fidler, M.; Dulub, O.; Schmid, M.; Diebold, U.; Hou, W.; Aschauer, U.; Selloni, A. (Sub)Surface Mobility of Oxygen Vacancies at the TiO 2 Anatase (101) Surface. *Phys. Rev. Lett.* **2012**, *109*.

(28) Enriquez, E.; Chen, A.; Harrell, Z.; Dowden, P.; Koskelo, N.; Roback, J.; Janoschek, M.; Chen, C.; Jia, Q. Oxygen Vacancy-Tuned Physical Properties in Perovskite Thin Films with Multiple B-Site Valance States. *Sci. Reports 2017 71* **2017**, *7* (1), 1–8.

(29) Xiao, C.; Zhou, Z.; Li, L.; Wu, S.; Li, X. Tin and Oxygen-Vacancy Co-Doping into Hematite Photoanode for Improved Photoelectrochemical Performances. *Nanoscale Res. Lett.* **2020**, *1* (54).

(30) Jubb, A. M.; Allen, H. C. Vibrational Spectroscopic Characterization of Hematite, Maghemite,

and Magnetite Thin Films Produced by Vapor Deposition. *ACS Appl. Mater. Interfaces* **2010**, *2* (10), 2804–2812.

(31) Barroso, M.; Mesa, C. A.; Pendlebury, S. R.; Cowan, A. J.; Hisatomi, T.; Sivula, K.; Grätzel, M.; Klug, D. R.; Durrant, J. R. Dynamics of Photogenerated Holes in Surface Modified α-Fe 2 O 3 Photoanodes for Solar Water Splitting. *Proc. Natl. Acad. Sci.* **2012**, *109* (39), 15640–15645.

(32) Hufnagel, A. G.; Hajiyani, H.; Zhang, S.; Li, T.; Kasian, O.; Gault, B.; Breitbach, B.; Bein, T.; Fattakhova-Rohlfing, D.; Scheu, C.; et al. Why Tin-Doping Enhances the Efficiency of Hematite Photoanodes for Water Splitting—The Full Picture. *Adv. Funct. Mater.* **2018**, *28* (52), 1804472.

(33) Zhang, Z.; Karimata, I.; Nagashima, H.; Muto, S.; Ohara, K.; Sugimoto, K.; Tachikawa, T. Interfacial Oxygen Vacancies Yielding Long-Lived Holes in Hematite Mesocrystal-Based Photoanodes. *Nat. Commun. 2019 101* **2019**, *10* (1), 1–12.

(34) Parka, H. J.; Hong, S. Y.; Chun, D. H.; Kang, S. W.; Park, J. C.; Leea, D.-S. A Highly Susceptive Mesoporous Hematite Microcube Architecture for Sustainable P-Type Formaldehyde Gas Sensors. *Sensors Actuators B Chem.* **2019**, *287*, 437–444.

(35) Wu, C.; Yin, P.; Zhu, X.; Ouyang, C.; Xie, Y. Synthesis of Hematite (r-Fe 2 O 3) Nanorods: Diameter-Size and Shape Effects on Their Applications in Magnetism, Lithium Ion Battery, and Gas Sensors. *J Phys Chem B* **2006**, *110* (36), 17806–17812.

(36) Guan, C.; Liu, J.; Wang, Y.; Mao, L.; Fan, Z.; Shen, Z.; Zhang, H.; Wang, J. Iron Oxide-Decorated Carbon for Supercapacitor Anodes with Ultrahigh Energy Density and Outstanding Cycling Stability. *ACS Nano* **2021**, *9* (5), 5198–5207.

(37) Le Formal, F.; Pendlebury, S. R.; Cornuz, M.; David Tilley, S.; Grä, M.; Durrant, J. R. Back Electron−Hole Recombination in Hematite Photoanodes for Water Splitting. *J. Am. Chem. Soc.* **2014**, *136* (6), 2564–2574.
